# Supramolecular Self-Assembled Nanostructures Derived from Amplified Structural Isomerism of Zn(II)−Sn(IV)−Zn(II) Porphyrin Triads and Their Visible Light Photocatalytic Degradation of Pollutants †

**DOI:** 10.3390/nano14131104

**Published:** 2024-06-27

**Authors:** Nirmal Kumar Shee, Hee-Joon Kim

**Affiliations:** Department of Chemistry and Bioscience, Kumoh National Institute of Technology, Gumi 39177, Republic of Korea; nirmalshee@gmail.com

**Keywords:** structural isomers, porphyrins, supramolecular self-assembly, nanostructures, photocatalytic degradation

## Abstract

Two structural isomeric porphyrin-based triads (Zn(II)porphyrin−Sn(IV)porphyrin−Zn(II)porphyrin) denoted as **T1** and **T2** were prepared from the reaction of *meso*-[5-(4-hydroxyphenyl)-10,15,20-tris(3,5-di-tert-butylphenyl)porphyrinato]zinc(II) (**ZnL**) with trans-dihydroxo-[5,10-bis(3-pyridyl)-15,20-bis(phenyl)porphyrinato]tin(IV) (**SnP^1^**) and trans-dihydroxo-[5,15-bis(3-pyridyl)-10,20-bis(phenyl)porphyrinato]tin(IV) (**SnP^2^**), respectively. All the compounds were characterized using UV–vis spectroscopy, emission spectroscopy, ESI−MS, ^1^H NMR spectroscopy, and FE-SEM. Most importantly, the two structurally isomeric porphyrin-based triads supramolecularly self-assembled into completely different nanostructures. **T1** exhibits a nanosphere morphology, whereas **T2** exhibits a nanofiber morphology. The amplified geometric feature in the structural isomeric porphyrin-based triads dictates the physical and chemical properties of the two triads. Both compounds showed the morphology-dependent visible light catalytic photodegradation of rhodamine B dye (74–97% within 90 min) and tetracycline antibiotic (44–71% within 45 min) in water. In both cases, the photodegradation efficiency of **T2** was higher than that of **T1**. The present investigation can significantly contribute to the remediation of wastewater by tuning the conformational changes in porphyrin-based photocatalysts.

## 1. Introduction

Well-shaped and highly ordered nano- and micromaterials have drawn significant appreciation from the material chemistry community owing to their vast applications in several areas such as solar energy conversion and storage [1], sensing [2], catalysis [3], biomedicine [4], and molecular recognition [5]. Additionally, these materials show interesting features, including high surface area [6], high thermal stability [7], excellent electrical conductivity [8], and excellent optoelectronic properties [9], compared to their starting molecules. Various building block components have been utilized to fabricate micro- and nanostructured materials [10,11,12,13,14,15,16]. Porphyrin molecules are the emerging building blocks for the formation of self-assembled functional nano- or micromaterials with well-defined dimensions [17,18,19,20,21]. In solids and solutions, porphyrin compounds self-assemble to form large-scale aggregates. Several non-covalent interactions (intermolecular), such as π-π stacking interactions, electrostatic interactions, ligand coordination, hydrogen bonding interactions, and hydrophilic and hydrophobic interactions, facilitate the self-assembly of porphyrins [22,23,24,25]. Occasionally, the irregular morphologies of these nanomaterials restrict their use in practical applications. Therefore, the design and fabrication of nanomaterials with suitable dimensions are challenging. Currently, Sn(IV)porphyrin-based molecules are extensively used as an ideal scaffold for the fabrication of supramolecular porphyrin nano-architectures that include nanotubes, nanosheets, nanofibers, nanorods, and nanocomposites [26,27,28,29,30]. Moreover, Sn(IV)porphyrin-based nanoaggregates have been widely used for photocatalytic applications, including photo-oxygenation [31], proton reduction [32], CO_2_-fixation [33], and organic dye degradation [34]. Sn(IV)porphyrin molecules can construct strong six-coordinated compounds with two trans oxyanions, either carboxylate or alkoxide, owing to the oxophilic nature of the Sn(IV) center. Sn(IV)porphyrin molecules are diamagnetic, and their structural details can be readily derived from NMR studies. Moreover, these compounds exhibit interesting structures and photochemical properties [35,36,37,38,39,40]. Several techniques have been described for the fabrication of well-defined porphyrin nanostructures, including surfactant-assisted methods [41], re-precipitation [42], metal–ligand coordination [43], sonication [44], and ionic self-assembly [45].

Previously, our group has reported multiple Sn(IV)porphyrin-based nanomaterials derived from the solvothermal reactions of trans-dihydroxo-[5,15-bis(3-pyridyl)-10,20-bis(phenyl)porphyrinato]tin(IV) **SnP^2^** with various Zn(II)porphyrins [46,47]. Intramolecular metal–ligand coordination between Zn(II)porphyrins and the pyridyl-N atom of Sn(IV)porphyrin, followed by π-π stacking interactions of porphyrin units in triads, was the crucial factor for the construction of these nanoaggregates. However, these studies were not limited to the 3-pyridyl positions but also extended to the 2-pyridyl and 4-pyridyl positions [48]. From these studies, it is clear that the [Zn(II)porphyrin−Sn(IV)porphyrin−Zn(II)porphyrin]-containing triads derived from **SnP^2^** show more uniform nanoaggregates than the other pyridyl positions.

**Scheme 1:**
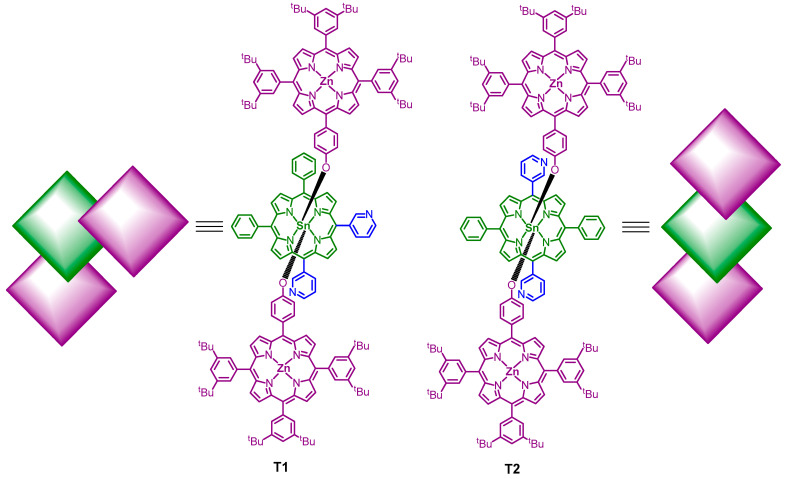
Chemical structures and alignments of the pair of isomeric triads discussed in the present study.

With this in mind, we examined the condensation of trans-dihydroxo-[5,10-bis(3-pyridyl)-15,20-bis(phenyl)porphyrinato]tin(IV) **SnP^1^** with *meso*-[5-(4-hydroxyphenyl)-10,15,20-tris(3,5-di-tert-butylphenyl)porphyrinato]zinc(II) **ZnL** and its self-assembly behavior. A new triad compound, **T1** (derived from the reaction of **ZnL** with **SnP^1^**), was used in this study (see Scheme 1). For comparison, another triad compound, **T2** (obtained from the reaction of **SnP^2^** with **ZnL**), was used. In terms of chemical structure, these two porphyrin-based triads can be considered a pair of structural isomers. The term structural or constitutional isomerism has been found in porphyrin chemistry [49,50,51]. The two Sn(IV)porphyrins present different orientations (*cis* for **SnP^1^** and *trans* for **SnP^2^**) with respect to the position of the 3-pyridyl group. The porphyrin-based triads such as **T1** and **T2** are formed through the intramolecular metal–ligand cooperative coordination of the axial Zn(II)porphyrin with the two 3-pyridyl groups of the central Sn(IV)porphyrin. **SnP^1^** and **SnP^2^** bridge **ZnL** molecules with an orthogonal alignment in **T1** and a linear alignment in **T2**. Therefore, the intramolecular cooperative coordination of the axial Zn(II)porphyrin with the central Sn(IV)porphyrin amplifies the structural isomerism of the central Sn(IV)porphyrin.

These porphyrin-based triads (**T1** and **T2**) were examined using various analytical and spectroscopic methods, including ^1^H-NMR spectroscopy, FT-IR spectroscopy, photoluminescence spectroscopy, ESI-MS spectrometry, and UV–vis spectroscopy. The morphologies of these triads were analyzed by FE-SEM analysis. The results suggest that the coordination behavior controls the morphology of the nanostructures derived from the two structural isomeric triads. The intramolecular cooperative coordination of the axial Zn(II)porphyrin with the two 3-pyridyl groups of the central Sn(IV)porphyrin, followed by π-π interaction between the adjoining porphyrin triad, is the deciding factor for the fabrication of nanostructures. Our focus is thus to relate the isomeric coordination mode to the morphology-dependent self-assembly behavior of these triads. In addition, these nanostructures can exhibit the morphology-dependent catalytic degradation of pollutants under visible light irradiation.

The catalytic activities of **T1** and **T2** were thus investigated for the visible light catalytic degradation of rhodamine B (RhB) dye and tetracycline (TC) antibiotics in the present study. RhB is an amino xanthene-class water-soluble synthetic dye. It is extensively used in the cosmetic, printing, leather, and textile industries as a synthetic coloring agent. Additionally, it is used as a fluorescent, biological stain, and food additive in several developing countries. It is one of the twenty polluted contaminants detected regularly in wastewater. It damages the eyes and skin and harms the respiratory, nervous, and reproductive systems. RhB is non-biodegradable and carcinogenic. Therefore, it is imperative for researchers to test the effectiveness of their as-prepared catalysts on the removal of this pollutant dye from wastewater [52,53,54,55,56,57,58,59,60]. On the other hand, TC is a readily available commercial antibiotic and has broad-spectrum antimicrobial activity. It is widely used to treat the plague, cholera, malaria, brucellosis, typhus, pneumonia, and syphilis. It is also used to cure Gram-positive and Gram-negative bacterial, chlamydia, and rickettsia infections and as a growth promoter in animals. It is one of the cheapest and most effective medicines and is on the World Health Organization list of vitally important medicines. TC is a highly water-soluble phenanthrene-core group-based antibiotic with a poor metabolic degradation rate. Therefore, the release of residues from this antibiotic and its metabolites into water systems contributes to an increase in resistance genes. Owing to the high presence of TC, drinking water has also become toxic to humans and has adverse side effects, such as vomiting, loss of appetite, diarrhea, poor dental development, rash, and kidney problems. Previously, TC has been employed as a model contaminant to verify the catalytic activity of the as-prepared catalyst [61,62,63,64,65,66,67,68,69,70,71]. In the present study, several experiments were performed to determine the optimal conditions for the visible-light catalytic decomposition of MB and TC pollutants in water. In addition, the kinetics and degradation mechanisms of the catalytic reactions were examined. These results provide an encouraging methodology for the synthesis of porphyrin-containing nanomaterials for wastewater treatment applications.

## 2. Materials and Methods

All the chemical compounds were purchased from commercial suppliers and used without further purification. The experiments were performed under a dry argon atmosphere using the standard Schlenk line method. Pyrrole was redistilled from a solution of calcium hydride, and toluene was obtained from a solution of sodium and benzophenone ketyl. **SnP^2^** and **ZnL** were prepared according to the previously published method [46]. Elemental analysis was performed using a ThermoQuest EA 1110 analyzer (Thermo Fisher Scientific, Waltham, MA, USA). ^1^H NMR spectra were obtained using a Bruker BIOSPIN/AVANCE III 400 spectrometer at 293 K (Bruker BioSpin GmbH, Silberstreifen, Rheinstetten, Germany). Electrospray ionization mass spectrometry (ESI-MS) was performed using a Thermo Finnigan Linear Ion Trap Quadrupole mass spectrometer (Thermo Fisher Scientific, Waltham, MA, USA). UV–vis spectra were recorded using a Shimadzu UV-3600 spectrophotometer (Shimadzu, Tokyo, Japan). Steady-state fluorescence spectra were recorded using a Shimadzu RF-5301PC fluorescence spectrophotometer (Shimadzu, Tokyo, Japan). Field-emission scanning electron microscopy (FE-SEM) images were obtained using a MAIA III (TESCAN, Brno, Czech Republic) instrument. Powder X-ray diffraction (PXRD) patterns were recorded using a Bruker AXS D8 Advance powder X-ray diffractometer (Bruker, Billerica, MA, USA). Fourier transform infrared (FT-IR) spectra (KBr) were recorded using a Shimadzu FTIR-8400S spectrophotometer (Shimadzu, Tokyo, Japan). The Brunauer–Emmett–Teller (BET) surface area was measured with an analyzer (BELSORP-mini volumetric adsorption equipment) using N_2_ adsorption isotherms at 77 K.

### 2.1. Synthesis of 5,10-bis(3-pyridyl)-15,20-bis(phenyl)porphyrin **H_2_P**

Mixed aldehyde condensation was used to prepare a free porphyrin base. Pyrrole (0.78 mL, 11.2 mmol, 4.0 eq.) was added to a mixture of 3-pyridinecarboxaldehyde (0.66 mL, 7.0 mmol, 2.5 eq.) and benzaldehyde (0.43 mL, 4.2 mmol, 1.5 eq.) in propionic acid (250 mL) and refluxed for 2 h. The solution was then incubated overnight. The solid material was filtered, rinsed with methanol, and dried in air. The desired porphyrin was separated by silica column chromatography (eluent: CHCl_3_/MeOH) to afford **H_2_P** (R*_f_* = 0.23 CHCl_3_/MeOH (98:2)). Subsequently, it was recrystallized from a mixture of acetonitrile/CHCl_3_ to give a purple powder. Yield: (0.070 g, 4%). Anal calculated for C_42_H_28_N_6_: C, 81.80; H, 4.58; N, 13.62. Found: C, 81.70; H, 4.80; N, 13.50. ^1^H-NMR (400 MHz, CDCl_3_, ppm): δ 9.46 (s, 2H, H2-Py), 9.08 (d, J = 5.3 Hz, 2H, H6-Py), 8.82–8.96 (m, 8H, *β*-pyrrole), 8.55 (d, J = 7.6 Hz, 2H, H4-Py), 8.22 (d, J = 6.8 Hz, 4H, H2,6-phenyl), 7.76 (m, 8H, H5-Py + *m*,*p*-Ph), −2.83 (s, 2H, NH). UV–vis(CHCl_3_): λ_nm_ (log ε), 417 (5.35), 515 (4.22), 550 (3.97), 591 (3.72), 645 nm (3.63). Photoluminescence (CHCl_3_, λ_nm_): 653, 719.

### 2.2. Synthesis of **SnP^1^**

In a typical process, **H_2_P** (0.05 g, 0.08 mmol) and SnCl_2_∙2H_2_O (0.21 g, 0.93 mmol) were added to 25 mL of pyridine and then refluxed for 10 h under constant stirring. Subsequently, the pyridine was decanted, and the residue was filtered using a celite pad after dissolution in CHCl_3_. The solvent was evaporated and dissolved in 20 mL of tetrahydrofuran. Approximately 10 mL of an aqueous solution of K_2_CO_3_ (0.22 g, 1.6 mmol) was mixed and refluxed for 10 h. THF was removed, and the solution was allowed to cool at 10 °C to allow the product to settle. The solid compound was filtered, washed with water, and oven-dried. Recrystallization (from CH_2_Cl_2_/acetonitrile) afforded 52 mg of the reddish compound. Yield (0.052 g, 85%). Anal calculated for C_42_H_28_N_6_O_2_Sn: C, 65.73; H, 3.68; N, 10.95; R, 19.64. Found: C, 65.50; H, 3.80; N, 10.60; R, 20.10. ^1^H NMR (400 MHz, CDCl_3_, ppm): δ 9.57 (s, 2H, H2-Py), 9.12-9.28 (m, 10H, *β*-pyrrole + H6-Py), 8.50 (d, *J* = 7.6 Hz, 2H, H4-Py), 8.28 (d, *J* = 6.8 Hz, 4H, H2,6-phenyl), 7.80-7.92 (m, 8H, *m*,*p*-phenyl + H5-Py). UV–visible (CHCl_3_): λ_nm_ (log *ε*), 426 (5.55), 519 (3.58), 557 (4.33), 597 (3.98). Photoluminescence (CHCl_3_, λ_ex_ = 550 nm): 610 nm and 655 nm.

### 2.3. Synthesis of triad 1 (**T1**) 

**SnP^1^** (0.038 g, 0.05 mmol) and **ZnL** (0.103 g, 0.1 mmol) were added to 10 mL of toluene (anhydrous) under an argon atmosphere and refluxed for 48 h. The solution was then cooled to room temperature (298 K), and 20 mL of *n*-hexane was added to the reaction mixture and stirred for 2 h. The solution was filtered through a reddish-brown precipitate and air-dried. Yield: (0.111 g, 80%). Anal calculated for C_178_H_176_N_14_O_2_SnZn_2_: C, 76.55; H, 6.35; N, 7.02; R, 10.08. Found: C, 76.27; H, 6.72; N, 7.10; R, 9.91. ^1^H NMR (400 MHz, DMSO-d_6_, ppm): δ 9.48 (s, 2H, H2-Py), 9.10–9.20 (m, 10H, central-{*β*-pyrrole + H6-Py}), 8.85 (m, 8H, axial-*β*-pyrrole), 8.66 (d, *J* = 8.2 Hz, 4H, axial-*β*-pyrrole), 8.55 (d, *J* = 8.2 Hz, 4H, axial-*β*-pyrrole), 8.45 (d, *J* = 7.6 Hz, 2H, central-H4-Py), 8.27 (d, *J* = 6.8 Hz, 4H, central-H2,6-phenyl), 8.05 (s, 12H, axial-*meso*-O-Ar), 6.60 (d, *J* = 8.5 Hz, 4H, *β*-bridging phenyl), 2.72 (d, *J* = 8.5 Hz, 4H, *α*-bridging phenyl), 7.70–7.85 (m, 14H, axial-H4-Ar + central-{H5-Py *+ m*,*p*-phenyl}), 1.50 (s, 108H, ^t^Bu). UV–vis (toluene): λ_nm_ (log *ε*), 428 (5.78), 564 (4.88), 612 (4.84). Photoluminescence (toluene, λ_nm_): 597 and 646.

### 2.4. Photoelectrochemical Measurement

The photocurrent response and electrochemical impedance spectra were obtained using an electrochemical workstation (CHI660D) with a Na_2_SO_4_ solution (0.1 M) as the electrolyte using a three-electrode system (saturated calomel, working, and platinum wire electrodes). The working electrode was fabricated as follows: **T1** and **T2** (5 mg each) were dispersed in EtOH (3.0 mL) by sonication, deposited on the indium tin oxide (ITO) surface, dried in air, and heated at 160 °C for 10 h. Photoelectrochemical experiments were performed under a 150 W xenon arc lamp as a visible light source.

### 2.5. Photocatalytic Degradation Experiment

The catalytic efficiencies of **T1** and **T2** were explored using the catalytic photodegradation of RhB and TC antibiotics in water. For the source of visible light, a 150 W xenon arc lamp (ABET Technologies, Old Gate Lane, Milford, CT, USA) was used with a UV cut-off filter. Approximately 50 mg of the catalyst was added to an aqueous solution of 100 mL of RhB dye (50 mg L^−1^) with constant stirring. Then, the mixture was kept in the dark for half an hour to obtain an adsorption–desorption equilibrium. Approximately 4 mL of the solution was collected after irradiation with light at regular intervals. The catalyst was separated from the reaction vessel by simple filtration. The exact amount of RhB was determined by calculating the absorbance at 554 nm with a visible spectrophotometer. TC solutions were prepared from TC hydrochloride with distilled water (100 mg L^−1^, pH = 6.0). The exact amount of TC in the solution was determined by calculating the optical absorbance at 372 nm.

## 3. Results and Discussion

### 3.1. Syntheses

**Scheme 2:**
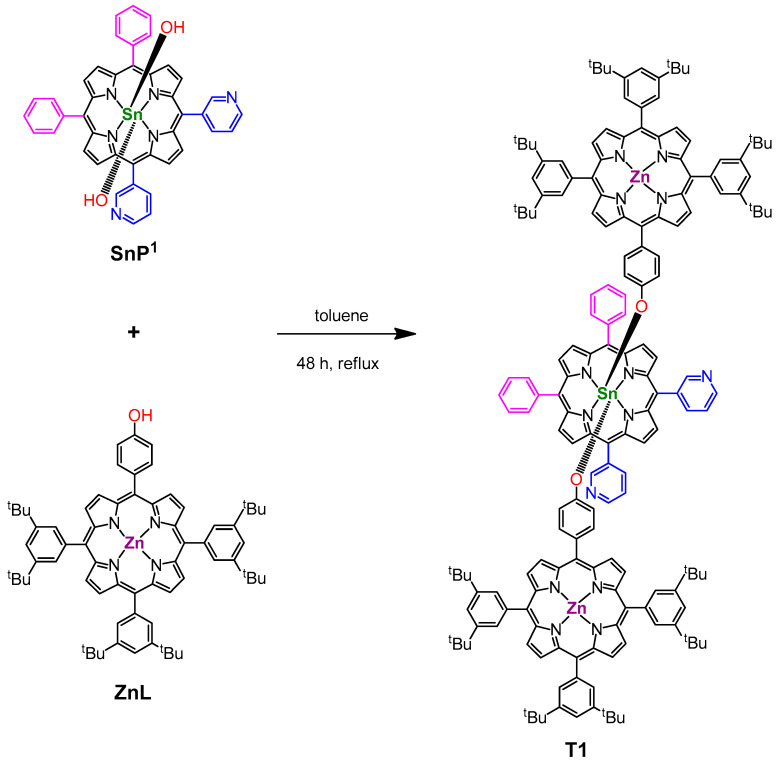
Synthesis of porphyrin-based triad **T1**.

The axial bonding approach has been utilized for the formation of porphyrin-containing triads **T1** or **T2** [43,46,47,48]. **T1** was prepared by refluxing one equivalent of **SnP^1^** and two equivalents of **ZnL** in dry toluene (Scheme 2). The complete procedure for synthesizing **SnP^1^** and **T1** was described in the Experimental section. The synthesis procedure and spectroscopic characterization of **SnP^2^**, **T2**, and **ZnL** were discussed in our previously published report [46]. The strong attraction of aryloxides towards Sn(IV)porphyrin was the impetus for the construction of these triads. Triads **T1** and **T2** were extensively analyzed using spectroscopic techniques such as elemental analysis, ESI-MS, FTIR, PXRD, ^1^H NMR, photoluminescence spectroscopy, UV–vis spectroscopy, and FE-SEM.

### 3.2. Spectroscopic Characterization

The ^1^H NMR spectra of **T1** and monomeric starting porphyrins (**H_2_L** and **SnP^1^**) are given in the Supplementary Materials (Figures S1−S3). ^1^H NMR spectra of **T2**, monomeric **ZnL**, and monomeric **SnP^2^** were discussed in a previously published report [46]. All peaks were assigned, and their integrations were indicated in the experimental section. The ^1^H NMR spectra of **T1** confirmed that the chemical shift and splitting patterns of the aromatic and *β*-pyrrolic protons of the central Sn(IV)porphyrin did not significantly differ from those of the starting monomer **SnP^1^**. The pyridyl protons at the 2-position of **T1** appeared as a singlet (9.48 ppm). Meanwhile, the *β*-pyrrolic protons and pyridyl protons at the 6-position of triad **T1** appeared in the range of 9.10–9.20 ppm (multiplate). The remaining protons (aromatic) of the central porphyrin (**SnP^1^**) of **T1** appeared in the range of 7.70–8.45 ppm.

Owing to the strong ring current effect of the central Sn(IV)porphyrin on the axial Zn(II)porphyrins, the protons of the axial Zn(II)porphyrins of **T1** differed from those of the starting **ZnL** in terms of resonance positions and splitting patterns. The aryloxy protons appeared at 7.19 ppm and 8.08 ppm in **ZnL** as a doublet. In triad **T1**, these two protons experienced a strong shielding effect from the ring current effect of the central Sn(IV)porphyrin and resonated at 2.72 and 6.60 ppm as a doublet. The Δδ values [i.e., δ(**ZnL**) − δ(triad **T1**)] for these protons were 4.47 and 1.48 ppm, respectively. Moreover, the *β*-pyrrolic protons of the axial Zn(II)porphyrins in triad **T1** exhibited a ring current effect similar to that of the starting **ZnL**. The *β*-pyrrolic proton resonated at 8.87 ppm in **ZnL**, whereas in **T1**, they were shifted to the upfield region and split into three various positions (8.55, 8.66, and 8.85 ppm). The remaining protons of the axial Zn(II)porphyrins emerged in the range of 7.70–8.05 ppm. These peaks did not change significantly with the composition of **ZnL**. Consequently, the ^1^H NMR technique was utilized to analyze the axial-bonding frameworks, which considers the interaction between the protons of the central Sn(IV)porphyrin with the protons of axial Zn(II)porphyrins and depicts the resonance couplings as well as ring-current-induced chemical shifts exclusively [43,46,47,48].

The ESI-MS spectrum of **T1** is shown in the Supplementary Materials (Figure S4). From the spectrum, it is clear that **T1** disintegrated during the mass spectrometry experiments. However, a peak at 2793.91 with a weak intensity appeared for the molecular ion [**T1**+H]^+^. The base peak at 768.42 is assigned to the fragment of [**SnP^1^**+H]^+^. Another major peak at 1031.74, corresponding to fragment [**ZnL**+H]^+^, was observed in the mass spectra.

**Figure 1:**
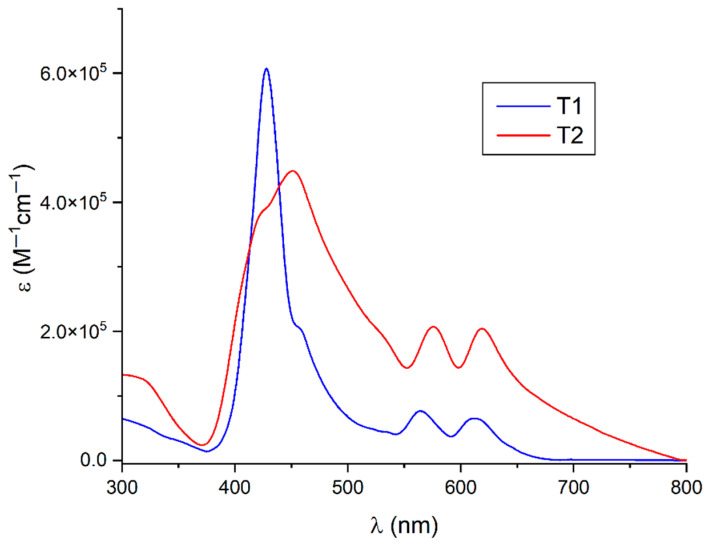
UV–vis spectra of triads **T1** and **T2** in toluene.

The UV–vis spectra of **T1** and **T2** in toluene are shown in Figure 1. The experimental section described all the spectral details, such as the peak positions at the maximum absorbance (λ_max_) and the molar extinction coefficient (ε). Zn(II) porphyrin **ZnL** showed a Soret band (419 nm) and Q-bands (547 and 586 nm). On the other hand, the monomeric Sn(IV)porphyrin **SnP^1^** exhibited a Soret band (426 nm) and Q-bands (519, 557, and 597 nm) [46]. The spectral features of **T1** and **T2** are distinct from those of the normal porphyrin-based triads. Generally, the magnitude of the molar extinction coefficient of the Zn(II)porphyrin−Sn(IV)porphyrin−Zn(II)porphyrin triad is similar to the total molar extinction coefficient of the starting porphyrins of Sn(IV)porphyrin and Zn(II)porphyrin [43,46,47,48].

Insignificant variation was observed for the triads (**T1** and **T2**) compared to their corresponding starting monomeric units, **SnP^1^**, **SnP^2^**, and **ZnL**. In the case of **T1**, peaks appeared in the Soret (428 nm) and Q-bands (564 and 612 nm). This implies that a small redshift and peak broadening occurred in the peaks of **T1** compared to those of either **SnP^1^** or **ZnL**. In the case of **T2**, the Soret band appeared at 455 nm, and the Q-bands appeared at 572 and 614 nm. These peaks were strongly red-shifted compared to those of **SnP^2^** or **ZnL**. In addition, the peaks of **T2** were broader than those of **T1.** These ground state results indicate that either there is no interaction or a low perturbation of the electronic structures of each π-conjugated porphyrin unit in **T1** compared to **T2**. In addition, basal–basal, basal–axial, or axial–axial couplings between the basal **SnP^1^** or axial **ZnL** were not observed so strongly for **T1** compared to **T2** [43,46,47,48]. Therefore, a peak broadening and a redshift of the peak position (λ_max_) of the Soret band indicates that a step-like or J-type arrangement is feasible for the self-aggregation of **T1** and **T2** in solution.

**Figure 2:**
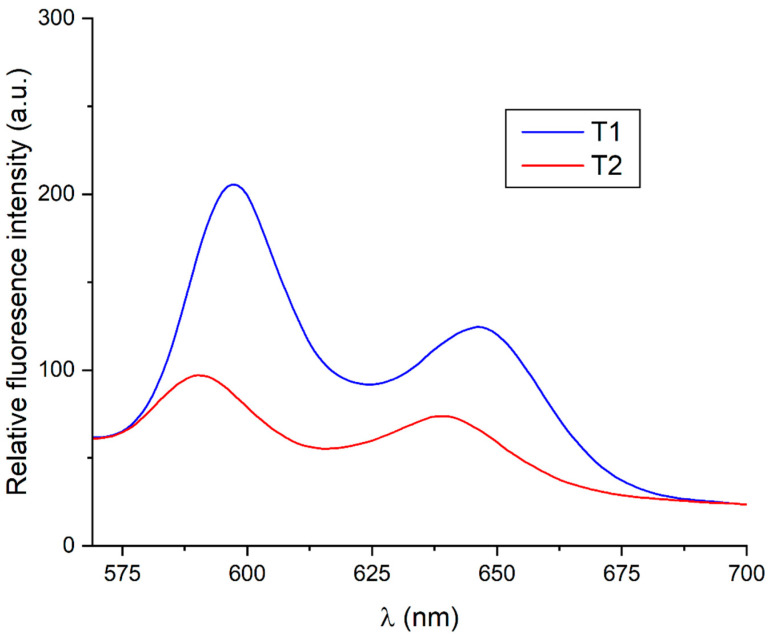
Photoluminescence spectra of **T1** and **T2** in toluene (λ*_ext_* = 550 nm). The optical density was fixed at 0.50.

The steady-state luminescence spectra of **T1** and **T2** are shown in Figure 2. **ZnL** exhibited two-banded photoluminescence spectra, with the emission peak (*λ*_max_) positioned at 600 and 645 nm. **SnP^1^** exhibited emission bands at 610 and 655 nm. **SnP^2^** exhibited emission bands at 609 and 656 nm. In the case of **T1**, the photoluminescence peaks appeared at 597 and 646 nm. In contrast, emission peaks appeared at 590 and 640 nm for **T2**. Both triads exhibited two-banded emission spectra with an alteration in the intensity ratio (peak-to-peak separation) compared to their corresponding monomeric starting porphyrin units. From Figure 2, it was confirmed that the emission intensity of both the triads is quenching, and their aggregation in solution directs the degree of quenching [43,46,47,48].

**Figure 3:**
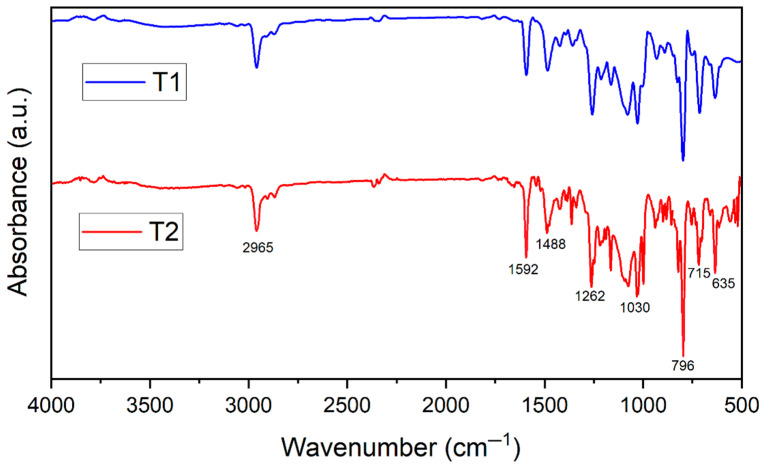
FT-IR spectra of **T1** and **T2**.

The FT-IR spectra of the triads **T1** and **T2** were recorded. Figure 3 clearly shows that both triads exhibit similar FT-IR spectra. The peak at 3600 cm^−1^ disappeared in both of the triads compared with the O-H stretching vibration of the hydroxyl group (axial) in the Sn(IV)porphyrins. The other peaks of the triads did not change compared with those of the starting monomeric porphyrin units.

**Figure 4:**
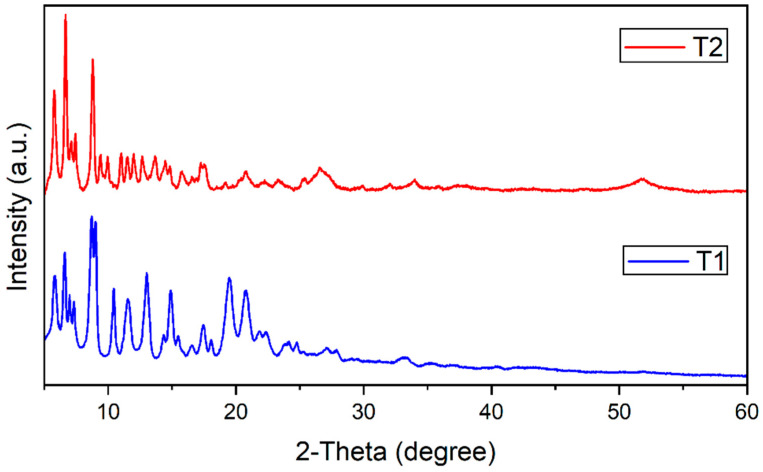
PXRD pattern of **T1** and **T2**.

To determine the crystalline nature of **T1** and **T2**, powder X-ray diffraction (PXRD) data for both triads were obtained, as shown in Figure 4. Figure 4 clearly shows that both **T1** and **T2** exhibit similar PXRD patterns, particularly for low-angle diffraction. **T1** shows major crystal peaks centered at 5.8°, 6.7°, 8.7°, 10.8°, 13.08°, 14.85°, and 19.55°. On the other hand, **T2** shows peaks at 5.8°, 6.7°, 8.7°, 9.4°, 9.9°, 11.0°, 11.6°, 12.0°, 12.6°, 13.7°, 14.4°, 17.4°, 20.6°, 26.5°, and 51.7°.

BET analysis was performed to determine the specific surface areas of both **T1** and **T2** (Figure S5). The nitrogen adsorption isotherms of **T1** are type I with a BET surface area of 63 m^2^/g, indicating a mesoporous network. On the other hand, the N_2_ sorption isotherms of **T2** showed a conjunction of type I and II isotherms with a BET surface area of 97 m^2^/g, confirming the coexistence of mesopores and micropores in the framework. This observation indicates that **T2** has a larger surface area compared to **T1**.

### 3.3. Microscopic Investigation

**Figure 5:**
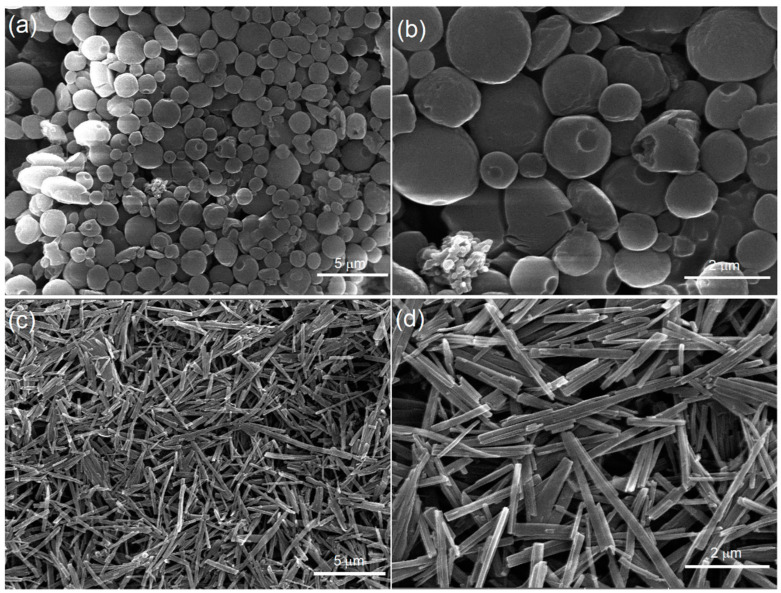
FE-SEM images of **T1** (**a**,**b**) and **T2** (**c**,**d**).

The self-assembly behavior and morphology of both triads were examined using FE-SEM. For the sample preparation, both triads were suspended in a mixed solvent (1:1 ratio of toluene to *n*-hexane) at a specified concentration of 0.5 mM. Then, the mixture solution was centrifuged for 10 s at 13,500 rpm and drop-cast on the surface of the Cu tapes, followed by air drying. After deposition, a platinum coating was applied before SEM analysis. Both **T1** and **T2** self-assembled into nanoaggregates, and their various morphologies are depicted in Figure 5.

Figure 5a,b clearly show that nanospheres of various sizes are observed for triad **T1**. The average diameter varied from 1500 to 1600 nm for the larger nanospheres. The average diameter varied from 780 to 820 nm for the smaller nanospheres. In contrast, well-defined nanofibers with homogeneous shapes and sizes were observed for **T2** (Figure 5c,d). The average length of the nanofibers varied from 2300 to 2700 nm. The average width of the nanofibers ranged from 160 to 190 nm. The aspect ratio amounts to approximately 14~15. The FE-SEM images of **T1** and **T2** show that all triad compounds self-assembled into nanostructures. Generally, free-base porphyrins or metalated porphyrins are self-assembled into nanoaggregates via *π*-*π* stacking interaction. Here, **T1** self-assembled via the *π*-*π* stacking interaction between the axial Zn(II)porphyrins and manifested spherical nanostructures. The orthogonal alignment of the axial Zn(II)porphyrins due to the coordination with the *cis* pyridyl groups of the central Sn(IV)porphyrin mainly contributed to the spherical morphology of **T1**. However, the morphology of **T2** completely differed from that of **T1**. **T2** exhibited well-defined nanofibers with good shape and dimension. The *trans* pyridyl groups of the central Sn(IV)porphyrin coordinate intramolecularly with the axial Zn(II)porphyrins, forming a linear arrangement of the axial Zn(II)porphyrins in **T2**. Therefore, the linear arrangement of **T2** readily promotes *π*-*π* stacking in a one-dimensional head-to-tail manner and subsequent self-assembly into nanofibers [46]. Notably, monomeric starting porphyrins such as **ZnL**, **SnP^1^**, and **SnP^2^** did not exhibit any significant nanostructured aggregation under the present experimental conditions [43,46,47,48].

### 3.4. Photocatalytic Degradation of Pollutants

**Figure 6:**
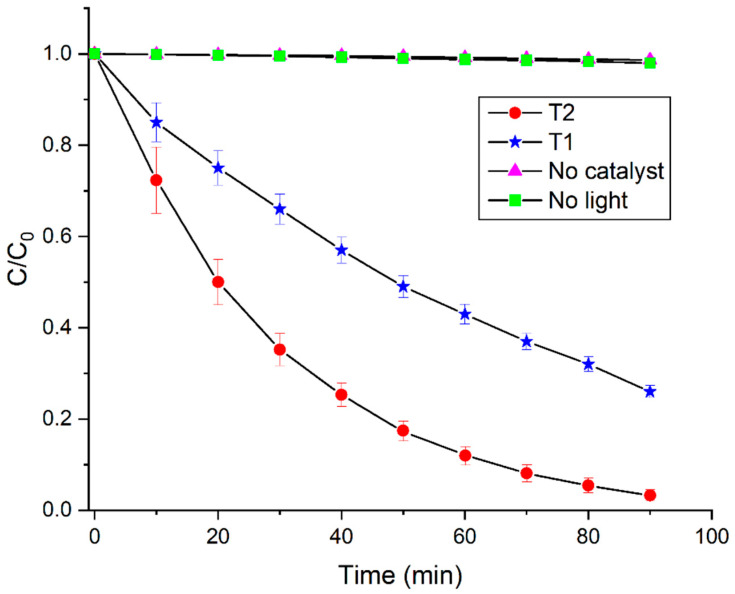
Visible light catalytic decomposition of RhB dye in water (pH = 7, temp = 298 K).

The photocatalytic efficiencies of **T1** and **T2** were examined by the visible light photodegradation of pollutants in water. RhB and TC were selected as target pollutants for photodegradation studies. As shown in Figure S6, 30 min was required to reach the adsorption–desorption equilibrium. **T1** adsorbed 14% of the RhB dye, whereas **T2** adsorbed 21%. The time-dependent optical absorption spectra of RhB in the presence of **T2** are shown in Figure S7. Without a catalyst or light source, only a small amount of RhB was decomposed (Figure 6). Therefore, catalysts and light were necessary for the photodegradation of RhB. The photodegradation of RhB was observed by calculating the absorbance peak at 554 nm as the irradiation time increased. The catalytic performance of both the triads towards the decay of RhB dye was demonstrated by using its decomposition efficiency, (C_0_−C)/C_0_, where C is the concentration at time t of the RhB dye and C_0_ is the initial concentration. In Figure 6, **T2** exhibits a higher efficiency for the photodegradation of RhB than **T1** within 90 min. The photodegradation ratio reached 97% and 74% for **T2** and **T1,** respectively. The photocatalytic performance of **T2** was superior to that of **T1**. Certain structural aspects, such as surface area and morphology, may contribute to differences in the photocatalytic performance. We now know the details of the reaction kinetics for the decay of RhB, allowing us to further elucidate our observations. For this, the pseudo-first-order model is demonstrated by the equation ln(C_0_/C) = kt, which is commonly used for catalytic photodegradation reactions if the initial concentration of the target contaminant is low, where k is the pseudo-first-order degradation rate constant. Based on the data in Figure 6, the plot of ln(*C*_0_/*C*) vs. *t* (time) was used to determine the photodegradation rate of the RhB dye (Figure S8). The first-order photodegradation rate constant for RhB by **T2** (0.037 min^−1^) is 2.5 times higher than that for **T1** (0.014 min^−1^). The above rate constants are promising when compared to the photodegradation rate constants of RhB dye using various catalysts under identical conditions (Table 1).

**Figure 7:**
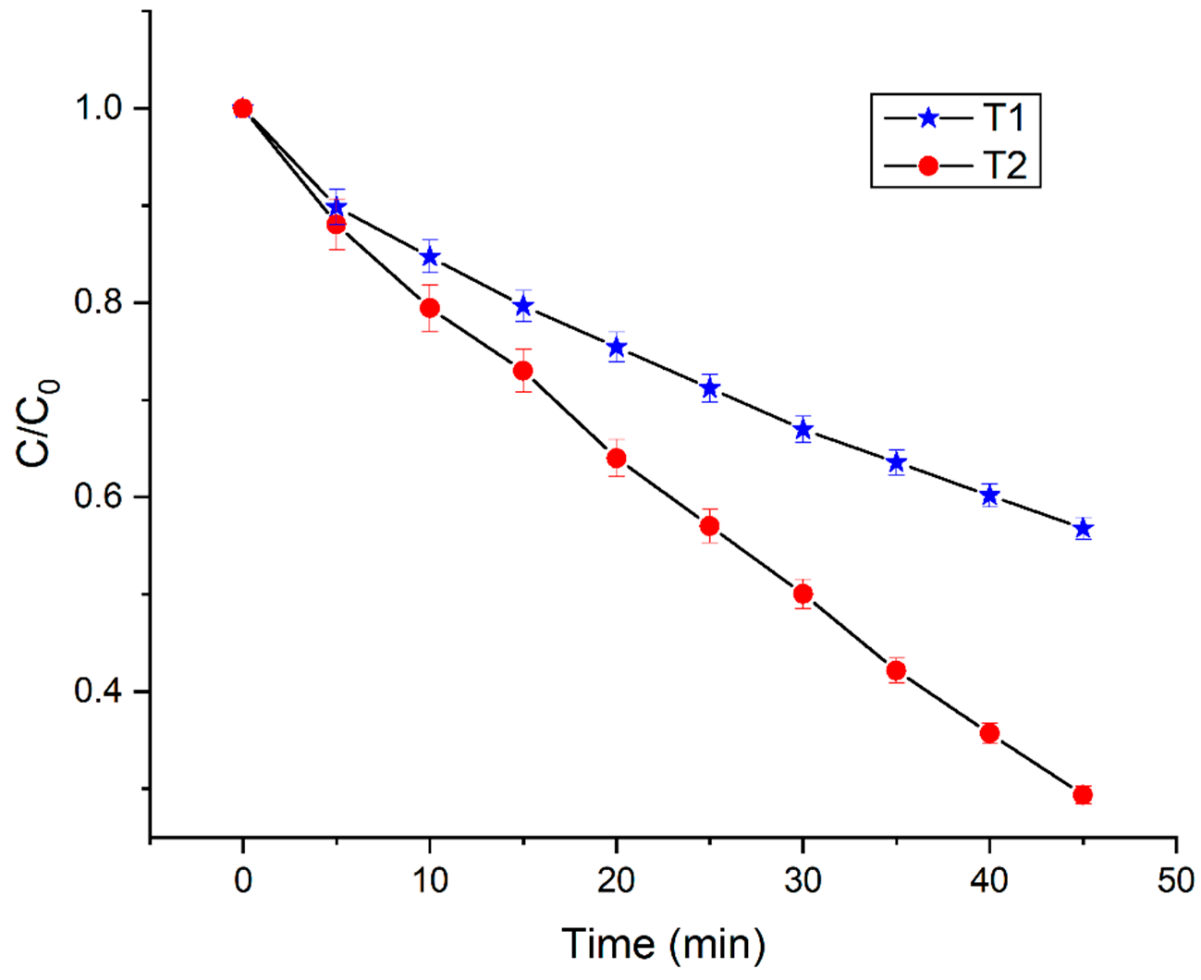
Visible light photodegradation of an aqueous solution of TC (pH = 6.0, temp = 298 K).

The universality of the photocatalytic degradation efficiency of **T1** and **T2** was further examined in the decomposition of TC. The time-dependent absorption spectra of TC in the presence of **T2** under visible light exposure are shown in Figure S9. As shown in Figure 7, the observed degradation ratios reached 71% and 44% for the photocatalysts derived from **T2** and **T1,** respectively, within 45 min. The pseudo-first-order degradation rate constant for the decomposition of TC by **T1** (0.012 min^−1^) is lower than that by **T2** (0.026 min^−1^) (Figure S10). Table 2 compares the rate constants for the photodegradation of TC with those of various reported catalysts.

From the above observations, it is clear that porphyrin-based triads **T1** and **T2** exhibit significant catalytic decomposition of water pollutants. Moreover, the recovery of the catalysts after the degradation reaction is very easy. After the completion of the reaction, the compounds were filtered, rinsed with H_2_O, and dried. The reusability of the photocatalyst is important for commercial applications, which was determined by recycling tests of **T2** for the RhB decomposition experiments (Figure S11). After 10 cycles, **T2** retained a high catalytic degradation performance of the RhB dye with a slight decrease (5%), indicating outstanding stability. In addition, the morphologies of **T1** and **T2** after the photodegradation reaction were similar to their initial FE-SEM images (Figure S12), confirming the high stability of these nanostructures during the course of the reaction. Figure S12 clearly shows that **T2** is more stable than **T1**. This observation also proves that nanostructure formation prevents hydrolysis of the photocatalyst during the degradation reaction. In addition, the FT-IR (Figure S13) and PXRD (Figure S14) spectra of **T1** confirmed the stability of the photocatalyst during the degradation reaction.

The optimum conditions for the decomposition reaction must be determined in terms of the dye/catalyst ratio, temperature, and pH of the dye solution. To this end, the decomposition reactions were performed at several temperatures to determine the effect of temperature on the reaction rate. The photodegradation performance improved with increasing temperatures up to 318 K (Figure S15). Subsequently, it decreased with increasing temperatures. It is likely that with increasing temperature (above 318 K), the axial porphyrins dissociate from the triad molecules. The pH of the RhB solution affected the photodegradation rate of RhB dye (Figure S16). Figure S16 confirms that the photodegradation rate increases from an acidic medium (pH = 2) to a neutral medium (pH = 7) and then decreases with increasing basicity. Moreover, the degradation rate is highly affected at an acidic pH compared to a basic pH. It is likely that the porphyrin triads are critical in highly acidic and basic solutions. To determine the effect of the RhB/catalyst ratio on the photodegradation of the RhB dye, different amounts of RhB dye solution (concentrations varying from 10 to 80 mg L^−1^) were used, along with a fixed quantity of photocatalyst **T2** (50 mg each time). The photodegradation rate of the RhB dye decreased with increasing concentrations of the RhB dye solution (Figure S17). Additionally, to determine the effect of light intensity on the RhB dye degradation rate by photocatalyst **T2**, various monochromatic light wavelengths were used (Figure S18). A variation in trend was observed in the wavelength-dependent degradation of RhB dye. Furthermore, the wavelength of the light source makes a remarkable contribution to solar energy harvesting and photodegradation capacity. **T2** still exhibited a little catalytic degradation performance even at λ > 700 nm.

**Figure 8:**
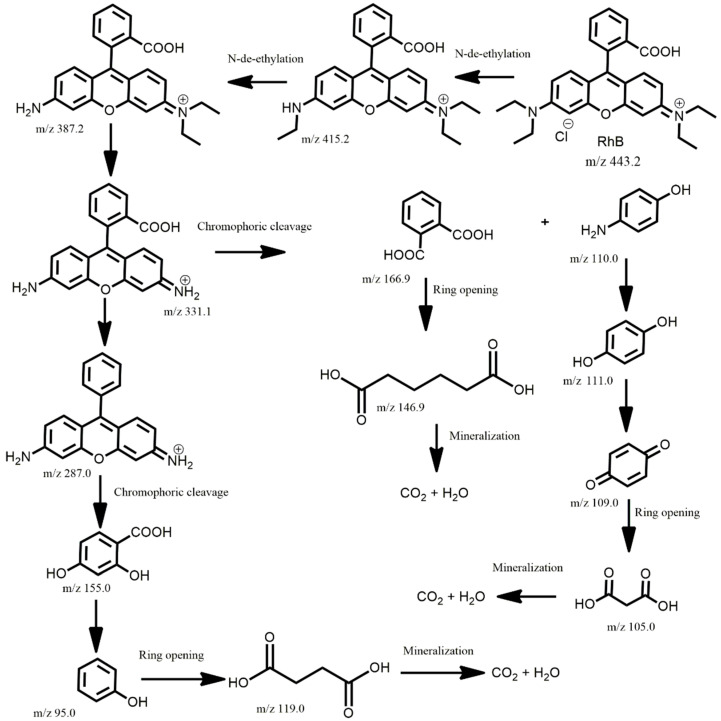
Possible intermediates for the RhB degradation in the presence of **T2** after 45 min of visible light irradiation.

To explore the complete details of RhB dye photodegradation products in the presence of **T2**, the reaction mixture was collected from the reaction vessels after 45 min and examined using ESI-MS (Figure S19). New peaks that develop in the mass spectra indicate the photodegradation of RhB into small, newer fragments [60]. Based on Figure S19, feasible intermediates for the photodegradation of RhB are shown in Figure 8. Initially, the base peak (*m*/*z* = 443.2) corresponded to the cationic form of RhB. RhB then underwent four successive *N*-de-ethylation reactions and fragmented into smaller parts with *m*/*z* = 415.2, 387.2, and 331.1. These cationic species disintegrated again after the rupture of the chromophoric group into smaller compounds with *m*/*z* values of 115.0, 166.9, and 110.0. These smaller aromatic molecules can sustain consecutive ring cleavages and hydrolysis, thereby generating low-molecular-weight compounds with *m*/*z* = 119.0 and 105.0. Finally, all intermediate fragments were decomposed and mineralized into CO_2_ and H_2_O. Additionally, the total organic carbon (TOC) removal percentage capacity for the degradation of RhB dye was estimated to be higher for **T2** (86%) than for **T1** (71%) for the degradation of RhB dye [60].

### 3.5. Possible Mechanism of the Photocatalytic Degradation Performance

Thus far, **T2** has shown better photocatalytic behavior against pollutants than **T1**. Before discussing the mechanism of the photocatalytic process, we examined this matter. Photocatalytic properties likely depend on factors such as the band gap energy, light harvesting properties, active surface area, and morphology of the photocatalyst. Moreover, the photogenerated charge separation, transportation, and lifetime of the reactive species control the photodegradation process. The band gap energy (E_g_) was calculated from Tauc’s plot using the absorption spectra [72]. The observed E_g_ of **T2** is 2.56 eV, which is lower than that of **T1** (2.72 eV) (Figure S20). Therefore, the lower band gap energy of **T2** indicates that it has better light-harvesting ability than **T1** and can utilize solar energy more effectively to generate more photogenerated carriers participating in photodegradation reactions. Since the separation efficiency of photogenerated carriers plays an important role in the photodegradation reaction, their behavior was investigated by fluorescence spectroscopy. As shown in Figure 2 (λ_ext_ = 550 nm), **T1** exhibits two-band photoluminescence spectra at 597 and 646 nm. On the other hand, **T2** exhibits a noticeable quenching and produces a blue shift to 590 and 640 nm, indicating the restricted recombination of photogenerated charge pairs (*h*^+^ and *e*^−^) and the intense interaction in the photocatalyst surface due to the electronic delocalization over the aromatic conjugated systems. 

Photoelectrochemical tests were performed to investigate the role of the charge-transfer complex in the catalytic photodegradation of **T2** and **T1**. When the light is on, the photocurrent of **T1** or **T2** first increases quickly and subsequently decreases, which may be due to the charge and discharge of the aromatic conjugated porphyrin layers during the polarization process of the electrode. As illustrated in Figure S21, **T2** displayed a better photocurrent response than **T1** (1.6 times higher than **T1**), suggesting that the photogenerated charge carrier separation of **T2** was significantly improved compared to **T1**. Furthermore, the arc radius of the electrochemical impedance spectroscopy (EIS) reflects the magnitude of the charge transfer resistance. Under visible light exposure, the EIS–Nyquist plot of **T2** exhibited a lower arc radius than **T1** (Figure S22), indicating that the working electrode had a smaller charge transfer resistance [73]. Therefore, the above observations indicate that **T2** is better than **T1** in terms of photogenerated charge transfer and carrier separation. The higher photocurrent and the lower resistance contribute to accelerated carrier separation and migration efficiency of **T2** compared to **T1**, which is advantageous for the photodegradation process.

**Figure 9:**
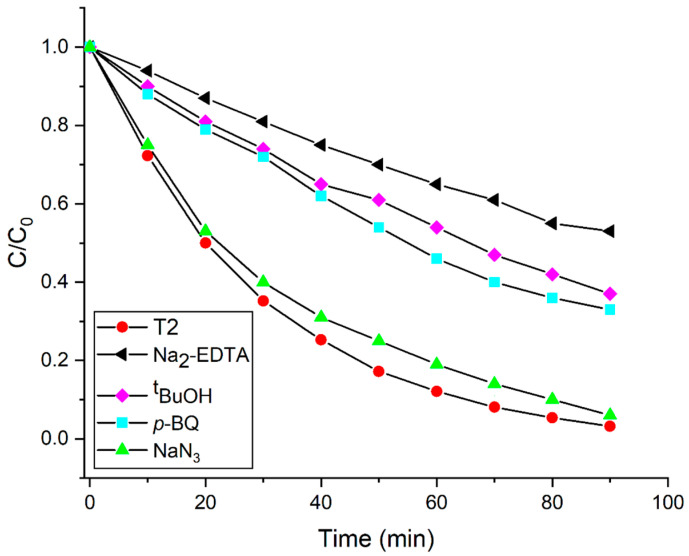
Visible light RhB degradation activities of **T2** in the presence of various scavengers.

Radical trapping experiments were also performed to understand the generation of photogenerated reactive species during the photodegradation reaction [74,75,76,77,78]. For this purpose, *para*-benzoquinone (*p*-BQ) was used to capture O_2_^−•^ (superoxide radical anion); *tert*-butanol (^t^BuOH) was used to apprehend ^•^OH (hydroxyl radical); sodium azide (NaN_3_) was used to capture singlet O_2_; and ethylenediaminetetraacetic acid disodium (Na_2_-EDTA) was utilized to detect photogenerated holes (h^+^) during the degradation experiment of RhB dye in the presence of **T2** (Figure 9). As shown in Figure 9, the degradation activity of **T2** was significantly affected by the presence of Na_2_-EDTA, ^t^BuOH, and *p*-BQ. However, the photodegradation of RhB was not affected by the presence of NaN_3_ or singlet oxygen. Initially, the degradation rate of RhB was found to be 97% without the influence of any scavengers. But, in the presence of Na_2_-EDTA, the degradation rate was drastically reduced to 48%. On the other hand, in the presence of ^t^BuOH or *p*-BQ, the photodegradation rate was found to be 63% and 67%, respectively. Hence, the above observation supports that the photogenerated holes (h^+^) are the principal reactive species that confer the photocatalytic degradation ability of **T2**.

Based on the above observations, a mechanism of carrier separation and photodegradation in the porphyrin-based triad photocatalyst (T) was proposed. After exposure to visible light, T can be excited and produce abundant hole–electron pairs (*h*^+^/*e*^−^) after crossing the band gap energy. Subsequently, the excited electrons trigger strong electronic delocalization over the surface of the aromatic conjugated systems in T and delay the recombination time. This promotes the generation of a large number of photogenerated charge pairs and boosts their transfer and separation from recombination. A highly reactive hydroxyl radical (^•^OH) is generated after the reaction of H_2_O with a photogenerated hole (*h*^+^), which degrades RhB into smaller molecules. The excited electrons react with O_2_ to generate superoxide radical anions (O_2_^−•^) and degrade the RhB dye into smaller compounds. Finally, all the smaller compounds are further mineralized into CO_2_ and H_2_O. In summary, the mechanism of the porphyrin-based triad catalyst (T) comprises five steps.

T + *hν* → T^∗^ (*e*^−^ + *h*^+^) (1)

O_2_ + *e*^−^ → O_2_^−•^ (2)

H_2_O + *h*^+^ → ^•^OH + H^+^ (3)

RhB + O_2_^−•^ → Degraded products (4)

RhB + ^•^OH → Degraded products (5)

Compared with the monomeric starting porphyrins, **T1** and **T2** showed significantly improved catalytic degradation abilities, which could be explained as follows: (i) **T1** or **T2** can remarkably improve the light absorption capacity in the solar light spectrum, thus producing more photogenerated carriers to assist in the photodegradation process. (ii) The rigid structure of **T1** or **T2** can strongly stabilize the photogenerated charge pairs over the conjugated aromatic systems, provide multidimensional channels for electron transfer, and delay the recombination time. (iii) Coordination-assisted self-assembled nanostructures in **T1** and **T2** provide multiple reaction sites and electron transfer pathways for the photocatalytic reaction. (iv) The permanent porosity of **T1** and **T2** with a high surface area permitted the enhanced adsorption of contaminants, thereby facilitating the photodegradation process. (v) More reactive species (^•^OH, O_2_^−•^, and *h*^+^) are produced in the photodegradation reaction, which can significantly enhance the degradation performance of **T1** or **T2**.

## 4. Conclusions

Two structural isomeric porphyrin-based triads (Zn(II)porphyrin−Sn(IV)porphyrin−Zn(II)porphyrin) were synthesized. The two Sn(IV)porphyrins have different orientations (*cis* for **SnP^1^** and *trans* for **SnP^2^**) with respect to the position of the 3-pyridyl group. In the porphyrin-based triad (**T1** and **T2**), the intramolecular cooperative coordination of the axial Zn(II)porphyrin with the two 3-pyridyl groups of the central Sn(IV)porphyrin occurs. Therefore, the structural isomerism of the central Sn(IV)porphyrin can be amplified by the intramolecular cooperative coordination in **T1** and **T2** to form an orthogonal alignment of Zn(II)porphyrins in **T1** and a linear alignment in **T2**, respectively. More interestingly, the two structurally isomeric porphyrin-based triads self-assembled into completely different nanostructures: nanosphere for **T1** and nanofiber for **T2**. The amplified geometric feature in the structural isomeric porphyrin-based triads dictates the physicochemical properties of these triads. Table 3 summarizes the physical and chemical properties of **T1** and **T2**. The present investigation has a significant impact on the treatment of dyeing wastewater by tuning the conformational changes in these porphyrin-based photocatalysts.

## Figures and Tables

**Table 1:** Comparison of the visible light photodegradation performance of RhB in the presence of various catalysts.

Photocatalyst	Rate Constant (min^−1^)	Reference
H_2_Ti_3_O_7_ nanotube	0.002	[52]
TiO_2_ (P-25)	0.001	[52]
Co_0.6_Zn_0.4_Fe_2_O_4_	0.015	[53]
TiO_2_	0.0015	[54]
TiO_2_/MgZnAl	0.005	[54]
ZnO	0.009	[55]
ZnO/Burkeite	0.028	[55]
Ni_0.5_Zn_0.5_Al_2_O_4_	0.005	[56]
SnO_2_-Acalypha Indica	0.0062	[57]
Au/ZnO	0.030	[58]
ZnS-NaBH_4_	0.0123	[59]
SnP	0.0016	[60]
TiO_2_	0.006	[60]
SnP@TiO_2_	0.0078	[60]
SnP/AA@TiO_2_	0.0366	[60]
**T1**	0.014	this study
**T2**	0.037	this study

**Table 2:** Comparison of the catalytic photodegradation performance of tetracycline (TC) with various catalysts under visible light irradiation.

Photocatalyst	Irradiation Time (min)	Catalyst Dosage (mg)	TC Concentration (mg/L)	Rate Constant (min^−1^)	Reference
CdS	60	10	40	0.0056	[61]
poly vinylidene fluoride-TiO_2_@g-C_3_N_4_-0.2g	300	100	50	0.012	[62]
SnO_2_	120	50	30	0.00324	[63]
g-C_3_N_4_	120	50	30	0.00473	[63]
3% SnO_2_/g-C_3_N_4_	120	50	30	0.0205	[63]
TiO_2_	100	25	10	0.0055	[64]
20 wt% MoS_2_/TiO_2_	100	25	10	0.0185	[64]
γ-In_2_Se_3_ (0.04 M EDTA)	120	50	20	0.0175	[65]
Fe_2_O_3_	120	50	10	0.00485	[66]
30%- Fe_2_O_3_/CuBi_2_O_4_	120	50	10	0.01246	[66]
La_2-x_Sr_x_NiMnO_6_ (x = 0.10)	240	100	10	0.0101	[67]
α-Fe_2_O_3_/g-C_3_N_4_	120	50	10	0.0222	[68]
WO_3_/g-C_3_N_4_/Bi_2_O_3_	60	100	10	0.0240	[69]
Ag_2_CO_3_/Ag/WO_3_	90	100	10	0.0179	[70]
WO_3_/g-C_3_N_4_	100	100	20	0.0210	[71]
**T1**	45	50	100	0.012	This study
**T2**	45	50	100	0.026	This study

**Table 3:** Comparison of the physical and chemical properties between two structural isomers **T1** and **T2**.

Structural Isomers	T1	T2
Starting materials	**ZnL + SnP^1^** (2:1)	**ZnL + SnP^2^** (2:1)
Chemical formula	C_178_H_176_N_14_O_2_SnZn_2_	C_178_H_176_N_14_O_2_SnZn_2_
Color	reddish-brown	dark green
Melting point	338−340 °C	346−348 °C
Solubility in toluene	highly soluble	sparingly soluble
Morphology (FE-SEM)	nanosphere	nanofiber
Self-assembly	intramolecular coordination for orthogonal alignment and intermolecular π-π interaction	intramolecular coordination for linear alignment and intermolecular π-π interaction
BET surface area (m^2^/g)	63	97
UV–vis absorption	428 nm (Soret band); 564, 612 nm (Q-band)	455 nm (Soret band); 572, 614 nm (Q-band)
Emission (excited at 550 nm)	597 nm and 646 nm	590 nm and 640 nm
Band gap energy (E_g_)	2.72 eV	2.56 eV
First-order degradation rate constant (RhB dye)	0.014 min^−1^	0.037 min^−1^
First-order degradation rate constant (TC antibiotic)	0.012 min^−1^	0.026 min^−1^

## Data Availability

Data are contained within the article or Supplementary Materials.

## Supplementary Materials

The following supporting information can be downloaded at https://www.mdpi.com/article/10.3390/nano14131104/s1. Figure S1. ^1^H NMR spectrum of 5,10-bis(3-pyridyl)-15,20-bis(phenyl)porphyrin **H_2_P** in CDCl_3_. Figure S2. ^1^H NMR spectrum of *trans*-dihydroxo-[5,10-bis(3-pyridyl)-15,20-bis(phenyl)porphyrinato]tin(IV) **SnP^1^** in CDCl_3_. Figure S3. ^1^H NMR spectrum of **T1** in DMSO-d_6_. Figure S4. ESI-mass spectrum of **T1**. Figure S5. Adsorption and desorption isotherms of N_2_ for **T1** and **T2** at 77 K. Figure S6. RhB dye adsorption test of **T1** and **T2**. Figure S7. Absorption spectra of RhB in the presence of **T2** under visible light irradiation. Figure S8. Kinetics of the photocatalytic degradation of RhB dye under visible light irradiation. Figure S9. Absorption spectra of TC in the presence of **T2** under visible light irradiation. Figure S10. Kinetics of the photocatalytic degradation of TC under visible light irradiation. Figure S11. Recyclability of the photocatalyst **T2** towards the degradation of RhB. Figure S12. FE-SEM images of **T1** and **T2** (after and before the degradation of RhB). Figure S13. FT-IR spectra **T1** (after and before the degradation of RhB dye). Figure S14. PXRD spectra **T1** (after and before the photodegradation of RhB). Figure S15. Effect of temperature on the degradation of RhB in the presence of **T2**. Figure S16. Effect of pH on the degradation of RhB solution in the presence of **T2**. Figure S17. Effect of dye concentration on the degradation of RhB in the presence of **T2**. Figure S18. Effect of wavelength dependence on the degradation of RhB in the presence of **T2**. Figure S19. Positive ion mode ESI-mass spectrum of the RhB degradation reaction by **T2** after 45 min of visible light irradiation. Figure S20. Band gap energy of **T1** and **T2** calculated from the Tauc’s plot using absorption spectral data. Figure S21. Photocurrent responses for **T1** and **T2** under visible light. Figure S22. EIS Nyquist plots for **T1** and **T2** under visible light.
