# Supplementary material for: Supramolecular Self-Assembled Nanostructures Derived from Amplified Structural Isomerism of Zn(II)−Sn(IV)−Zn(II) Porphyrin Triads and Their Visible Light Photocatalytic Degradation of Pollutants
Source: Nanomaterials (Basel). 2024 Jun 27;14(13):1104. doi: 10.3390/nano14131104 (PMC11243107; doi:10.3390/nano14131104)
Supplement: Supplementary file 1 [file nanomaterials-14-01104-s001.zip › nanomaterials-3052135-supplementary.pdf]

Supplementary Materials

Supramolecular Self-Assembled Nanostructures Derived from Amplified Structural Isomerism of Zn(II)–Sn(IV)–Zn(II) Porphyrin Triads and Their Visible Light Photocatalytic Degradation of Pollutants

Nirmal Kumar Shee and Hee-Joon Kim*

Department of Chemistry and Bioscience, Kumoh National Institute of Technology

61 Daehak-ro, Gumi 39177, Republic of Korea

List of contents:

Figure S1. ^{1}H NMR spectrum of 5,10-bis(3-pyridyl)-15,20-bis(phenyl)porphyrin **H_2P** in $CDCl_3$.

Figure S2. ^{1}H NMR spectrum of *trans*-dihydroxo-[5,10-bis(3-pyridyl)-15,20-bis(phenyl)porphyrinato]tin(IV) **SnP^1** in $CDCl_3$.

Figure S3. ^{1}H NMR spectrum of **T1** in DMSO-d_6.

Figure S4. ESI-mass spectrum of **T1**.

Figure S5. Adsorption and desorption isotherms of N_2 for **T1** and **T2** at 77 K.

Figure S6. RhB dye adsorption test of **T1** and **T2**.

Figure S7. Absorption spectra of RhB in the presence of **T2** under visible light irradiation.

Figure S8. Kinetics of the photocatalytic degradation of RhB under visible light irradiation.

Figure S9. Absorption spectra of TC in the presence of **T2** under visible light irradiation.

Figure S10. Kinetics of the photocatalytic degradation of TC under visible light irradiation.

Figure S11. Recyclability of the photocatalyst **T2** towards the degradation of RhB.

Figure S12. FE-SEM images of **T1** and **T2** (after and before the degradation of RhB).

Figure S13. FT-IR spectra **T1** (after and before the degradation of RhB).

Figure S14. PXRD spectra **T1** (after and before the degradation of RhB).

Figure S15. Effect of temperature on the photocatalytic degradation of RhB in the presence of **T2**.

Figure S16. Effect of pH on the degradation of RhB solution in the presence of **T2**.

Figure S17. Effect of dye concentration on the photocatalytic degradation of RhB in the presence of **T2**.

Figure S18. Effect of wavelength dependence on the photocatalytic degradation of RhB in the presence of **T2**.

Figure S19. Positive ion mode ESI-mass spectrum of the RhB degradation reaction by **T2** after 45 min of visible light irradiation.

Figure S20. Band gap energy of **T1** and **T2** calculated from the Tauc's plot using absorption spectral data.

Figure S21. Photocurrent responses for **T1** and **T2** under visible light.

Figure S22. EIS Nyquist plots for **T1** and **T2** under visible light.

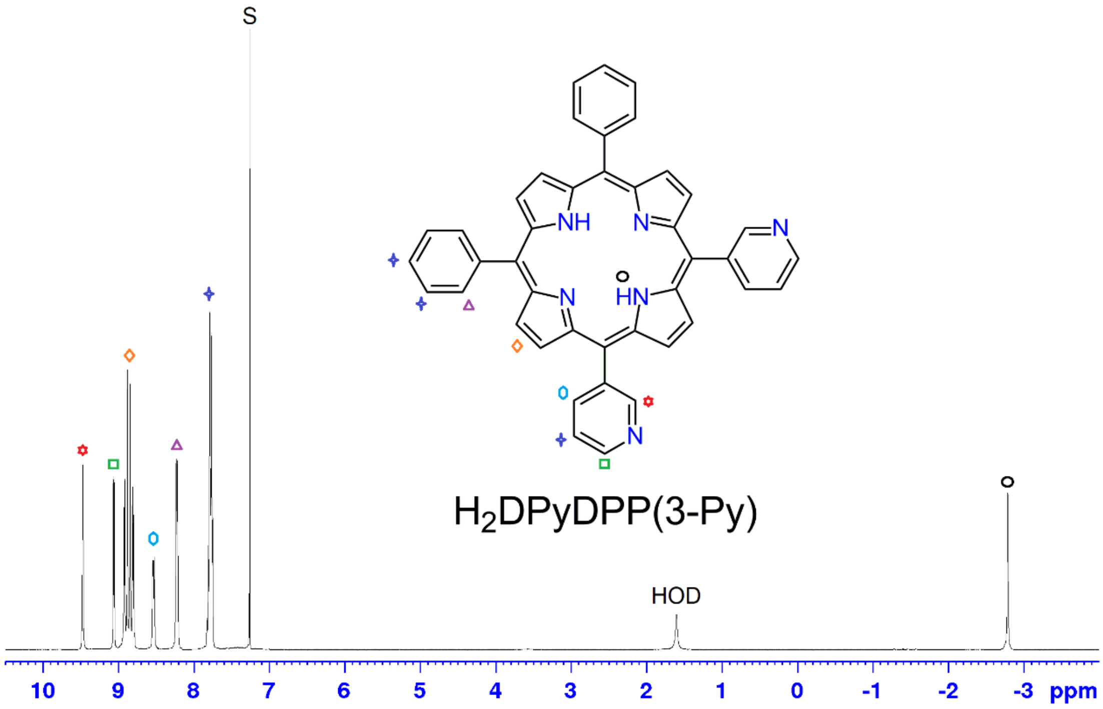

Figure S1. ^{1}H NMR spectrum of 5,10-bis(3-pyridyl)-15,20-bis(phenyl)porphyrin **H_2P** in $CDCl_3$.

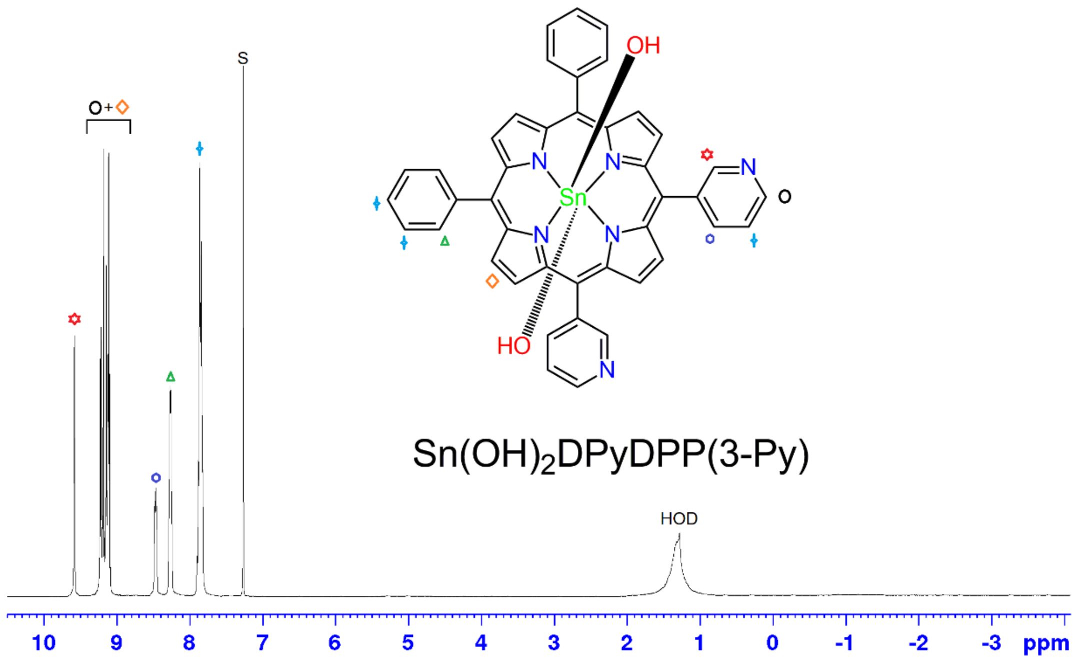

Figure S2. ^{1}H NMR spectrum of *trans*-dihydroxo-[5,10-bis(3-pyridyl)-15,20-bis(phenyl)porphyrinato]tin(IV) **SnP^1** in $CDCl_3$.

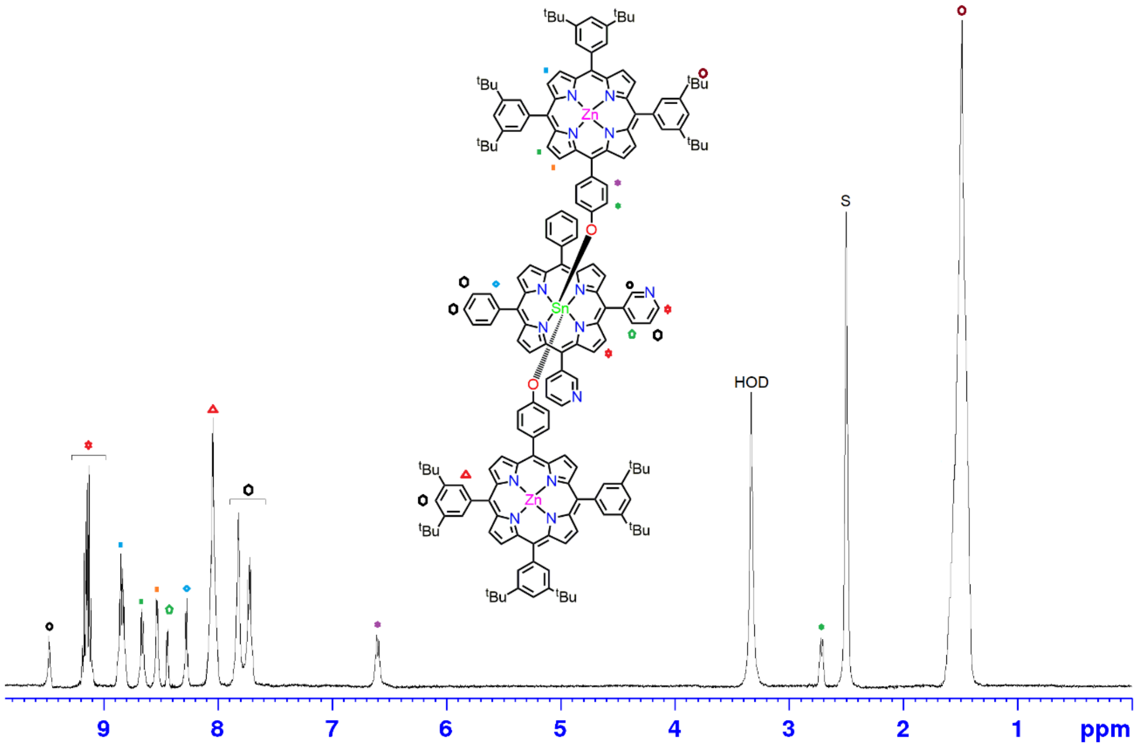

Figure S3. ^{1}H NMR spectrum of **T1** in DMSO-d$_6$.

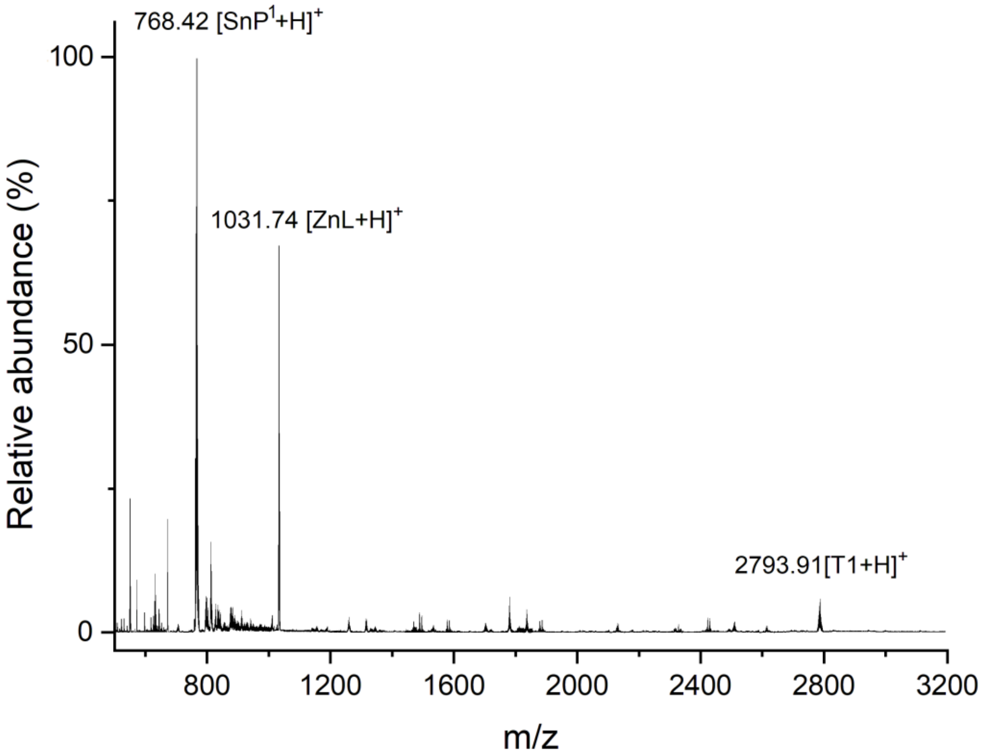

Figure S4. ESI-mass spectrum of **T1**.

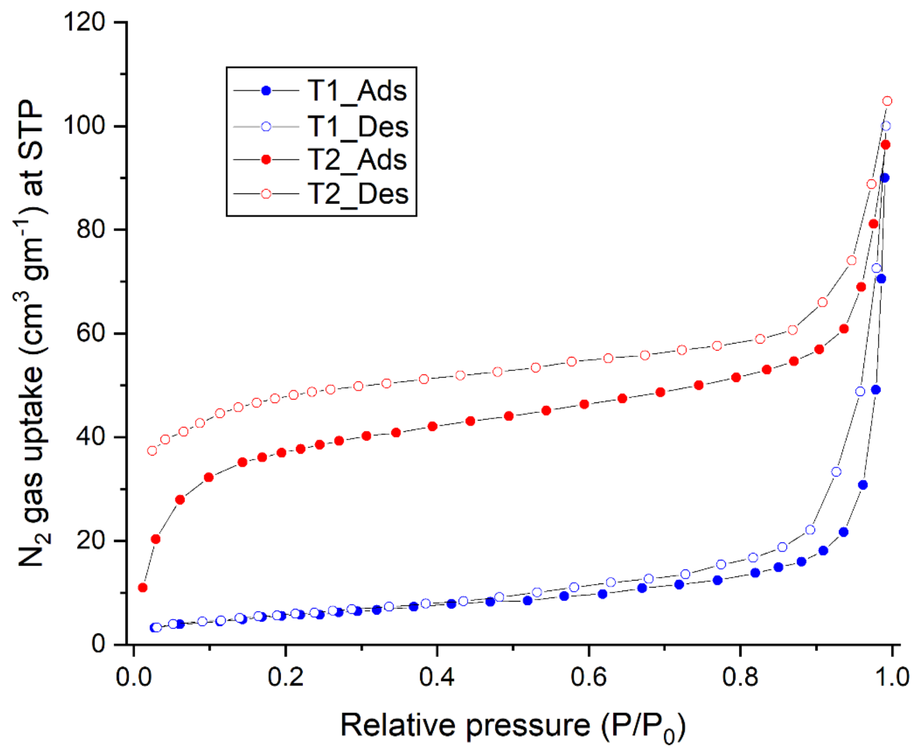

Figure S5. Adsorption and desorption isotherms of N_2 for **T1** and **T2** at 77 K.

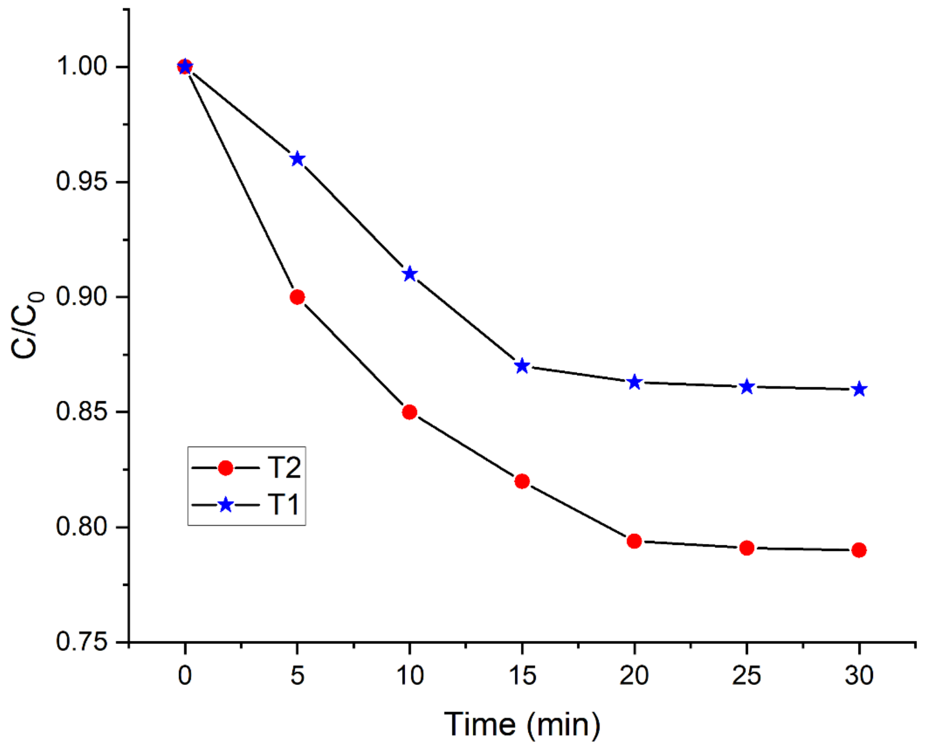

Figure S6. RhB dye adsorption test of **T1** and **T2**.

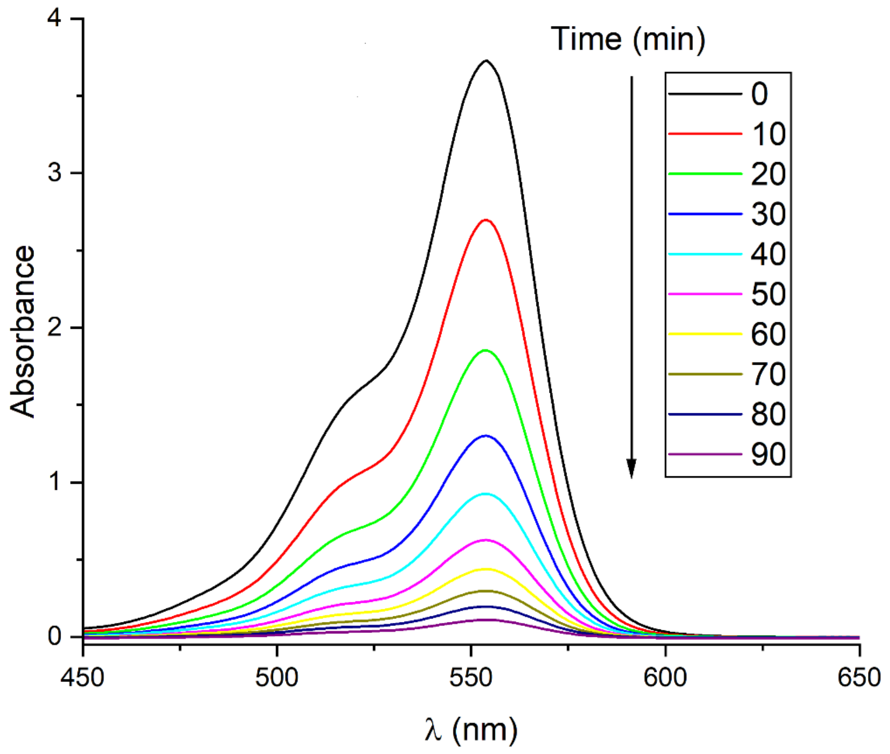

Figure S7. Absorption spectra of RhB in the presence of **T2** under visible light irradiation.

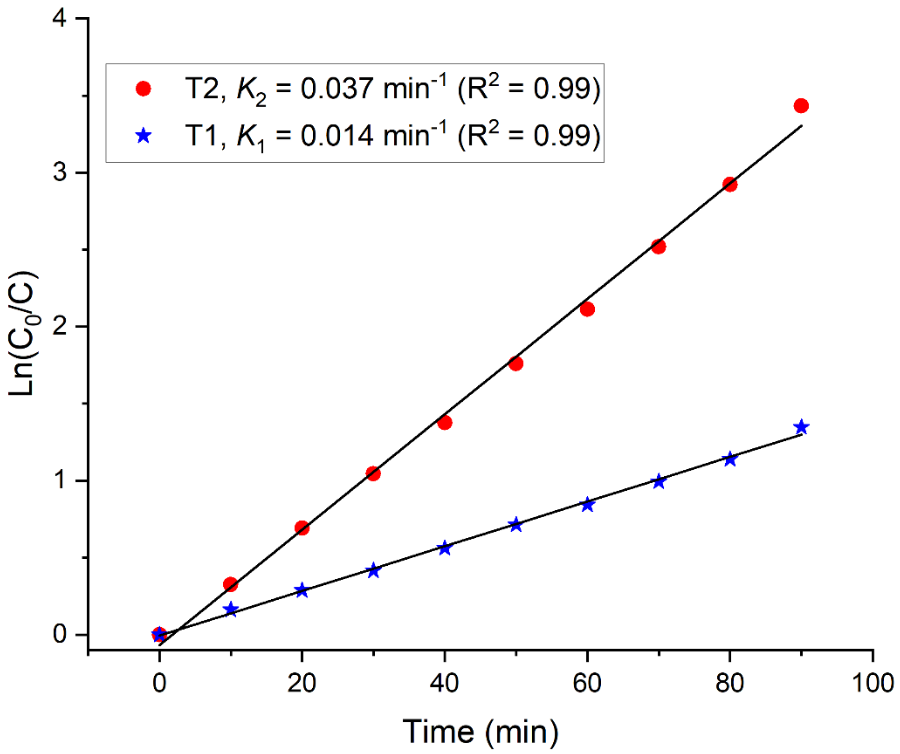

Figure S8. Kinetics of the photocatalytic degradation of RhB under visible light irradiation.

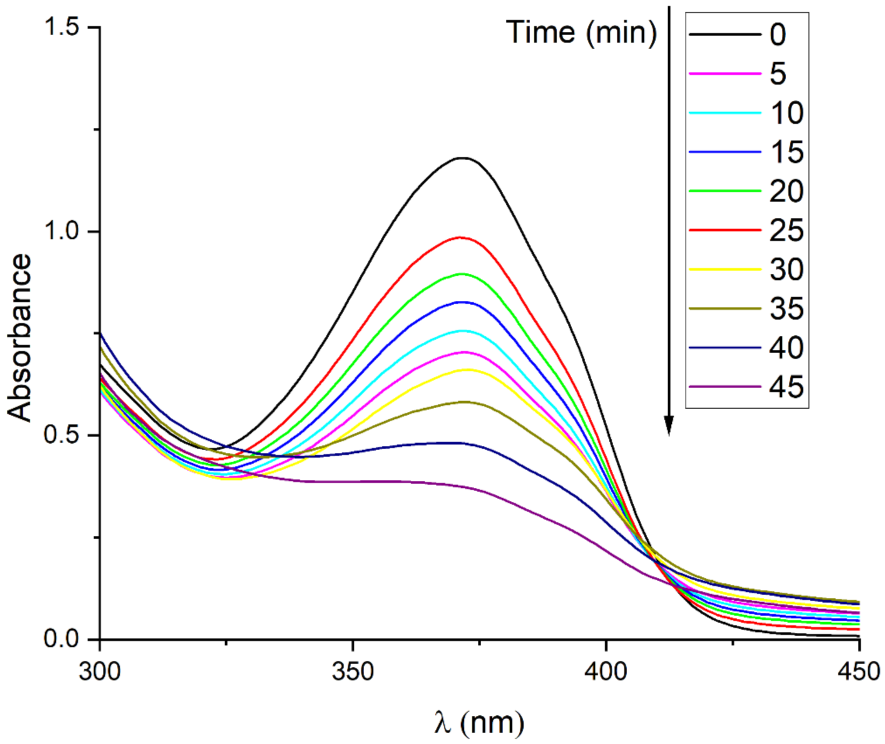

Figure S9. Absorption spectra of TC in the presence of **T2** under visible light irradiation.

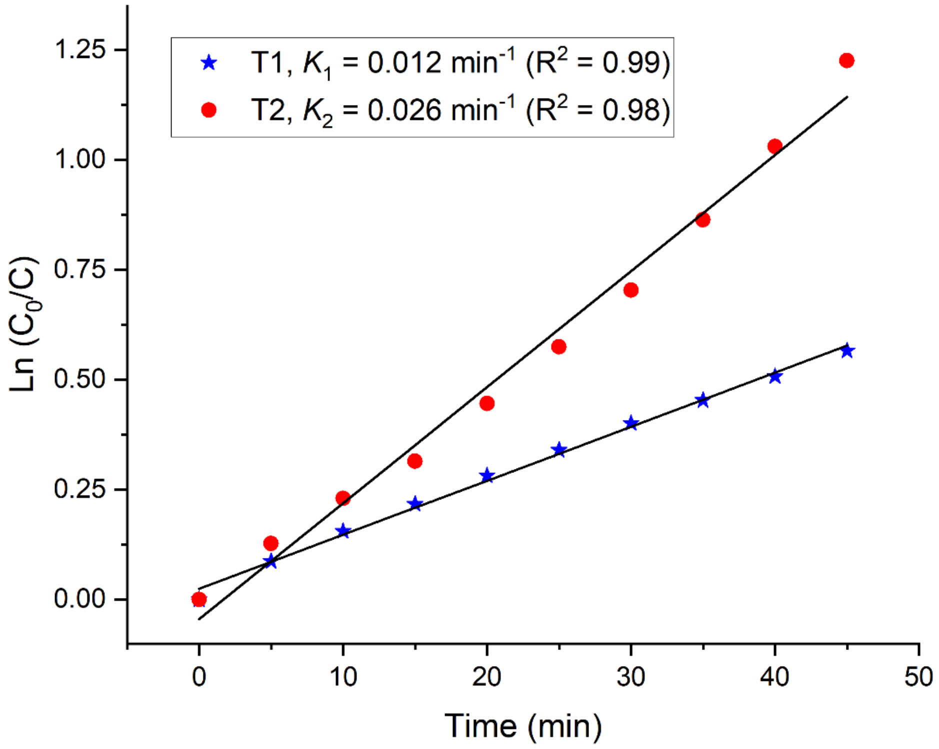

Figure S10. Kinetics of the photocatalytic degradation of TC under visible light irradiation.

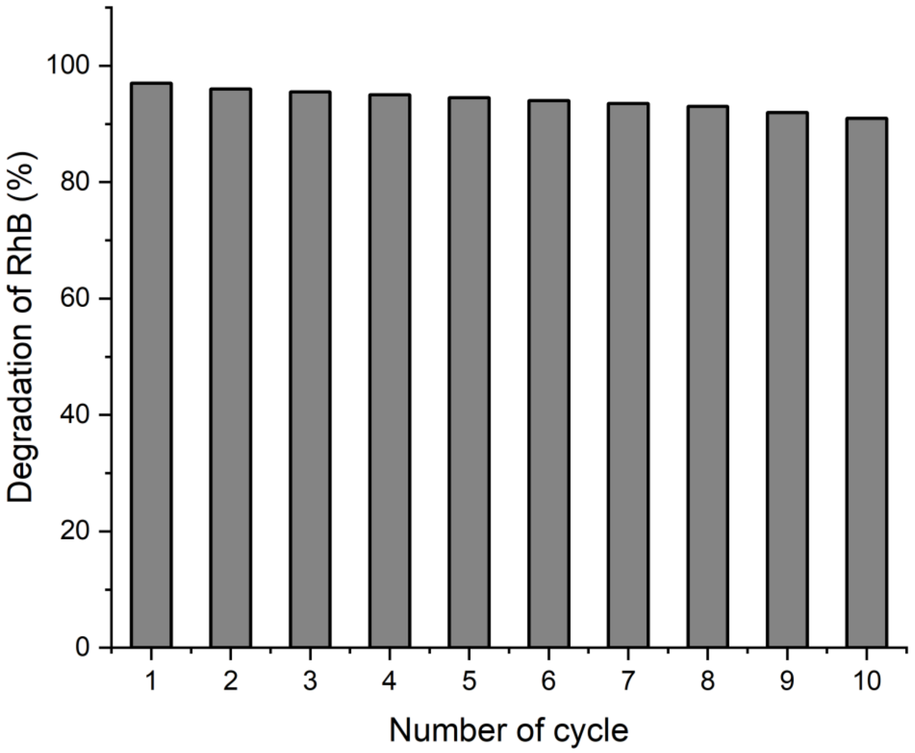

Figure S11. Recyclability of the photocatalyst **T2** towards the degradation of RhB.

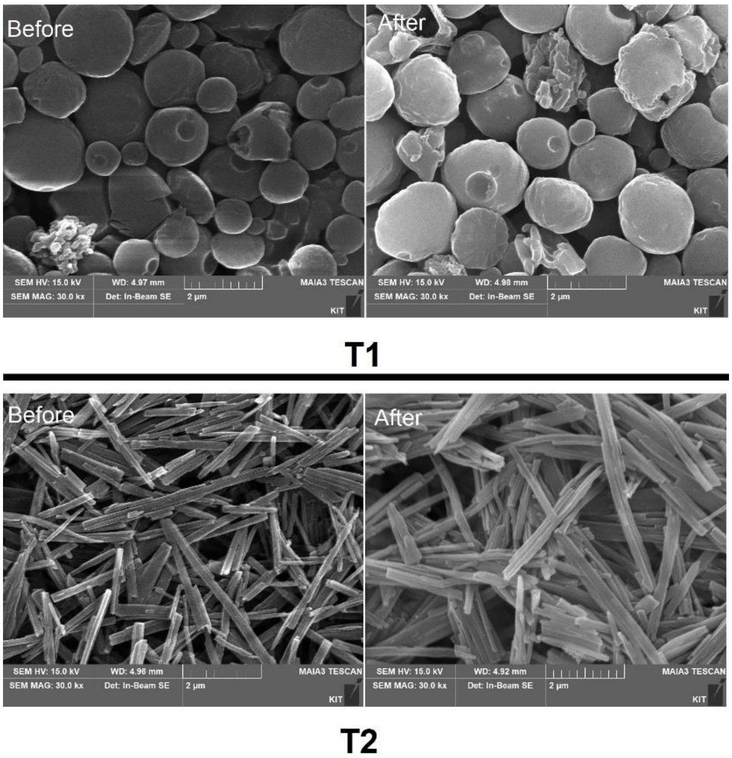

Figure S12. FE-SEM images of **T1** and **T2** (after and before the degradation of RhB).

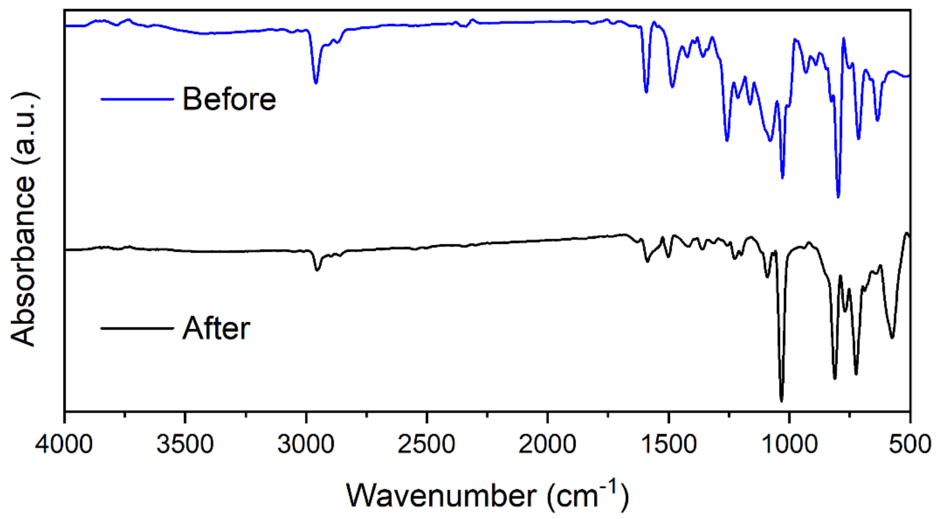

Figure S13. FT-IR spectra **T1** (after and before the degradation of RhB).

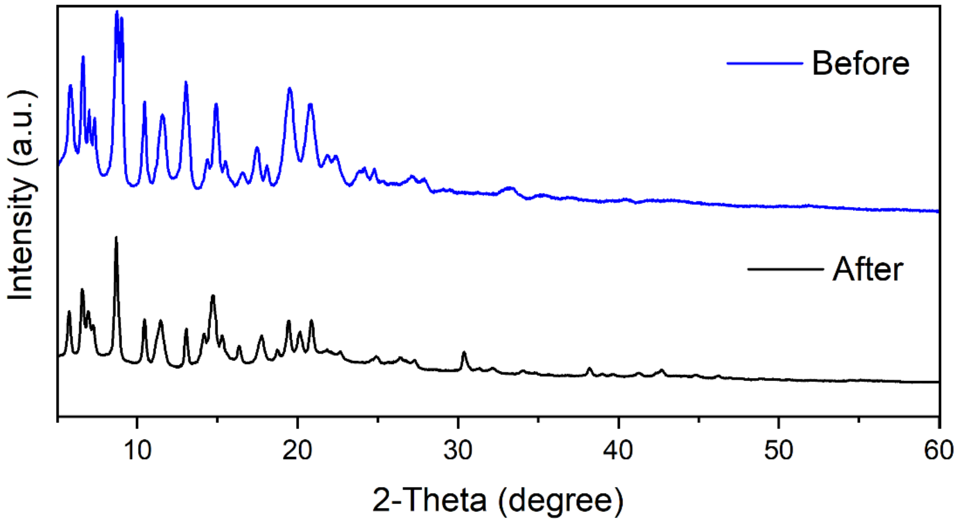

Figure S14. PXRD spectra **T1** (after and before the degradation of RhB).

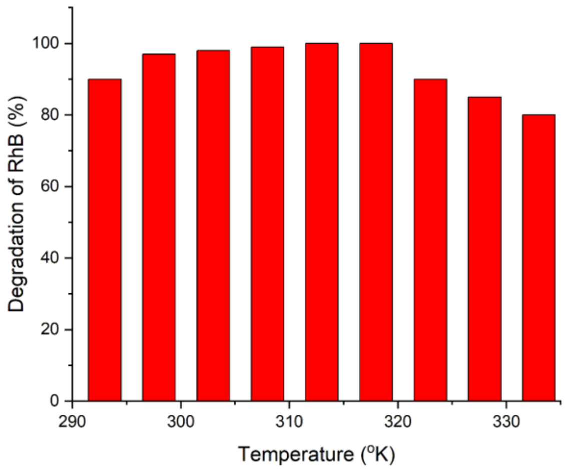

Figure S15. Effect of temperature on the photocatalytic degradation of RhB in the presence of **T2**.

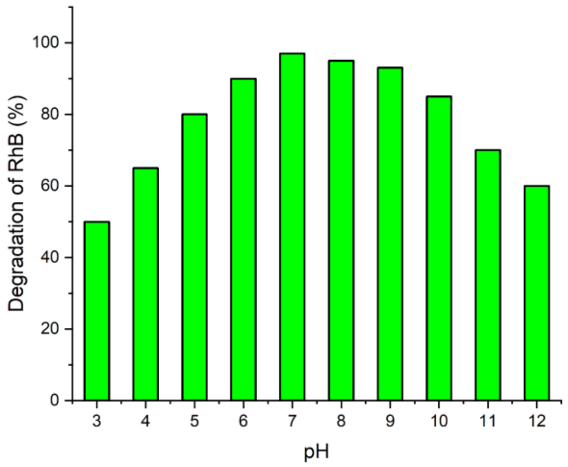

Figure S16. Effect of pH on the degradation of RhB solution in the presence of **T2**.

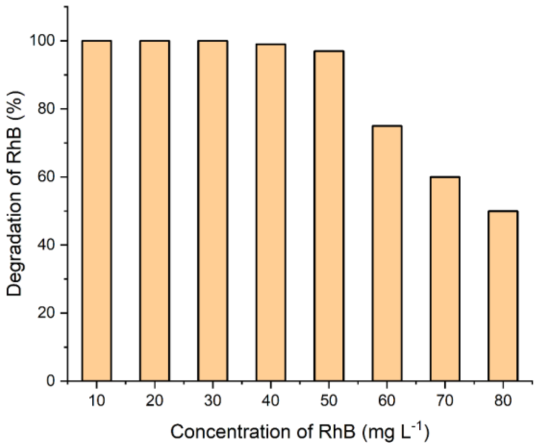

Figure S17. Effect of dye concentration on the photocatalytic degradation of RhB in the presence of **T2**.

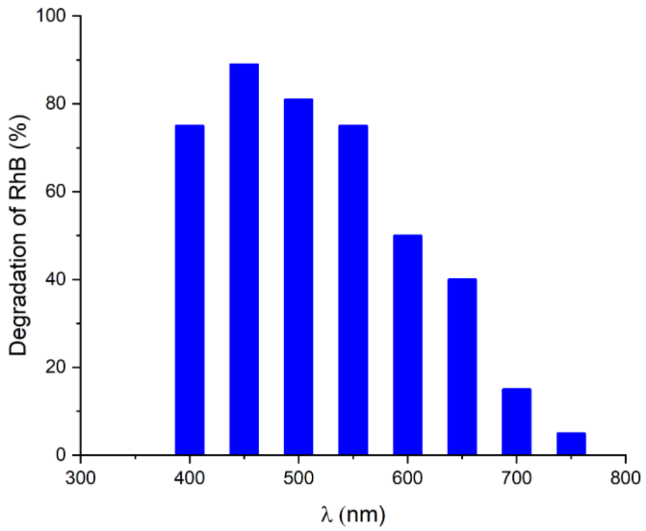

Figure S18. Effect of wavelength dependence on the photocatalytic degradation of RhB in the presence of **T2**.

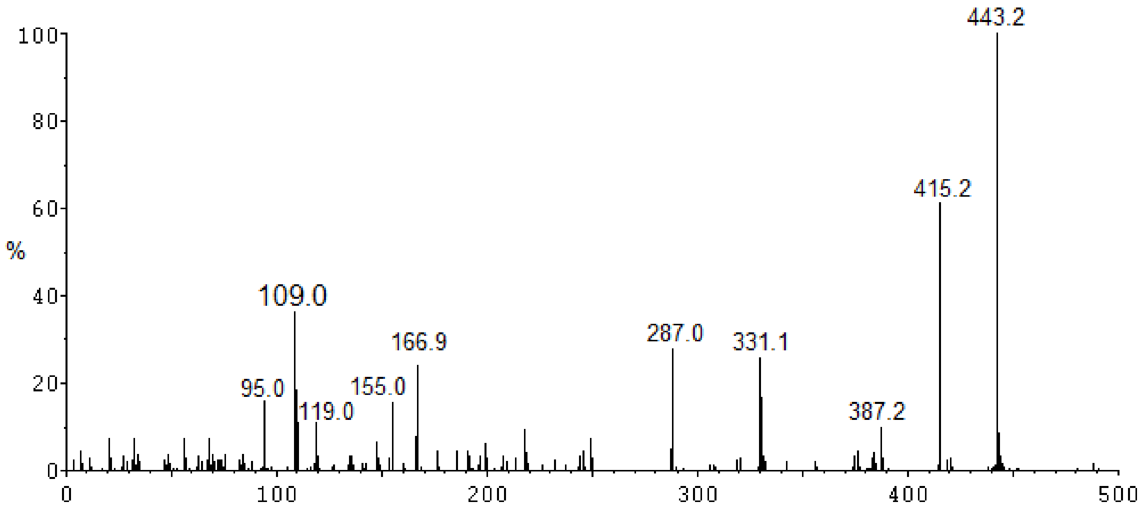

Figure S19. Positive ion mode ESI-mass spectrum of the RhB degradation reaction by **T2** after 45 min of visible light irradiation.

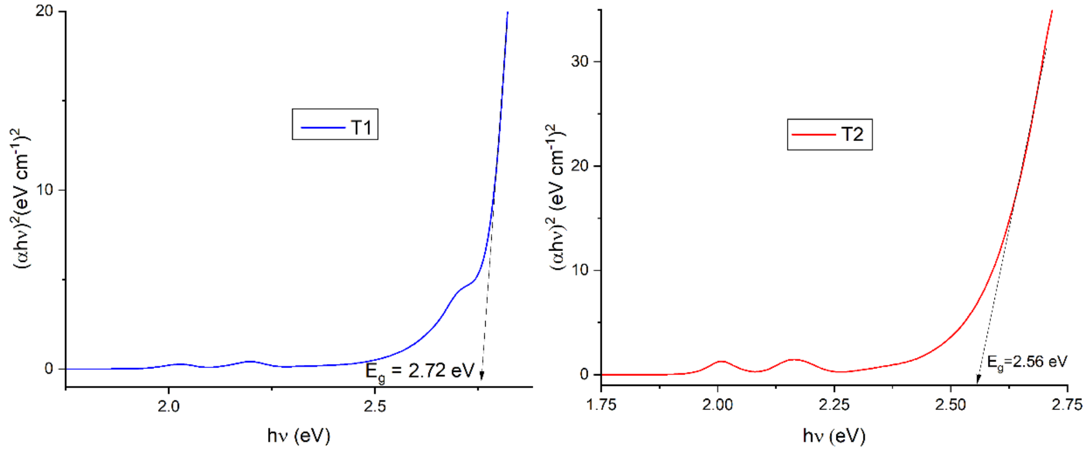

Figure S20. Band gap energy of **T1** and **T2** calculated from the Tauc's plot using absorption spectral data.

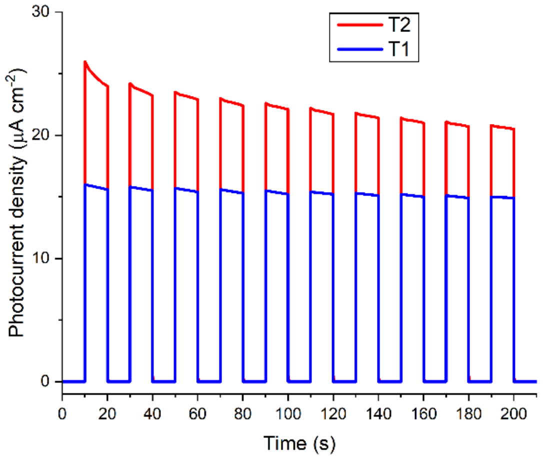

Figure S21. Photocurrent responses for **T1** and **T2** under visible light.

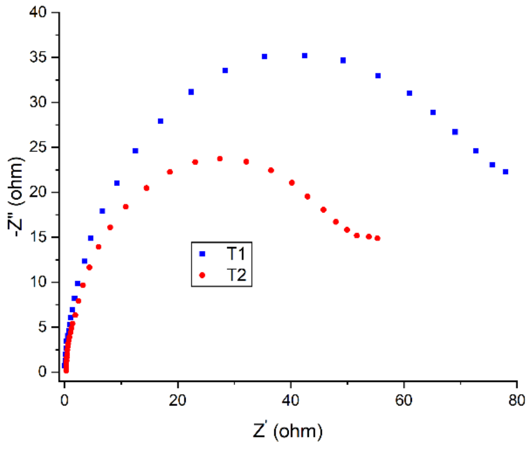

Figure S22. EIS Nyquist plots for **T1** and **T2** under visible light.
